# Unique molecular signatures of antiviral memory CD8^+^ T cells associated with asymptomatic recurrent ocular herpes

**DOI:** 10.1038/s41598-020-70673-z

**Published:** 2020-08-14

**Authors:** Swayam Prakash, Soumyabrata Roy, Ruchi Srivastava, Pierre-Gregoire Coulon, Nisha R. Dhanushkodi, Hawa Vahed, Allen Jankeel, Roger Geertsema, Cassandra Amezquita, Lan Nguyen, Ilhem Messaoudi, Amanda M. Burkhardt, Lbachir BenMohamed

**Affiliations:** 1 Laboratory of Cellular and Molecular Immunology, Gavin Herbert Eye Institute, School of Medicine, University of California Irvine, Hewitt Hall, Room 2032; 843 Health Sciences Rd, Irvine, CA 92697 USA; 2 Department of Molecular Biology and Biochemistry, School of Biological Sciences, University of California Irvine, Irvine, CA 92697 USA; 3 University Laboratory Animal Resources, University of California Irvine, Irvine, CA 92697 USA; 4 Vaccine Research and Development Center, Department of Physiology & Biophysics, University of California, Irvine, CA 92617 USA; 5 Institute for Immunology, School of Medicine, University of California Irvine, Irvine, CA 92697 USA

**Keywords:** Adaptive immunity, Immune evasion, Immunotherapy, Infection, Infectious diseases, Vaccines

## Abstract

The nature of antiviral CD8^+^ T cells associated with protective and pathogenic herpes simplex virus type 1 (HSV-1) infections remains unclear. We compared the transcriptome, phenotype, and function of memory CD8^+^ T cells, sharing the same HSV-1 epitope-specificities, from infected HLA-A*0201 positive symptomatic (SYMP) vs. asymptomatic (ASYMP) individuals and HLA-A*0201 transgenic rabbits. Compared to higher frequencies of multifunctional effector memory CD8^+^ T_EM_ cells in ASYMP individuals, the SYMP individuals presented dysfunctional CD8^+^ T_EM_ cells, expressing major exhaustion pathways. Compared to protected ASYMP HLA transgenic rabbits, the trigeminal ganglia of non-protected SYMP HLA transgenic rabbits had higher frequencies of dysfunctional tissue-resident CD8^+^ T_RM_ cells. Moreover, blockade of T cell exhaustion pathways restored the function of CD8^+^ T cells, reduced virus reactivation, and diminished recurrent disease in HLA transgenic rabbits. These findings reveal unique molecular signatures of protective CD8^+^ T cells and pave the way for T-cell-based immunotherapy to combat recurrent ocular herpes.

## Introduction

Herpes simplex virus type 1 (HSV-1), known to cause a wide range of diseases throughout an individual’s lifetime, is carried by a staggering 3.72 billion individuals worldwide^1^. Following ocular or oro-facial acute infection, HSV-1 enters the axon of sensory neurons that innervate the eyes and the mouth mucocutaneous tissues, and travel in a retrograde fashion to neuronal cell bodies of the trigeminal ganglion (TG), which becomes the site for HSV latency^2–8^. Physical, chemical, and emotional stressors trigger virus reactivation from latently infected TG, which then sheds in tears, and can cause a spectrum of recurrent ocular diseases^9–12^. Fortunately, virus shedding in tears is not harmful in the majority of seropositive asymptomatic (ASYMP) individuals^13^. However, in a minority of seropositive symptomatic (SYMP) individuals, the virus shedding in tears and re-infection of the eyes can develop into serious recurrent ocular diseases, including potentially blinding herpetic keratitis^14–26^.

Crosstalk between HSV-1 and CD8^+^ T cells restrains virus reactivation within the latently-infected TG^2,3,5,6,27^. The adaptive immunity plays a role in establishing latency as a high number of activated CD8^+^ T cells expressing a late effector memory phenotype were found to reside in latently infected TG. This indicates that activated late effector memory CD8^+^ T cells may control HSV-1 latency^7^. Functional antiviral CD8^+^ T cells, specific to multiple HSV-1 epitopes, are selectively retained and patrol latently-infected TG^2,5,6^. In contrast, dysfunctional HSV-specific CD8^+^ T cells appeared unable to control virus reactivation from latently infected TG, thus contributing to frequent virus shedding in tears as well as to frequent and severe recurrent herpes in SYMP individuals^5,28^. The process of partial or total impairment of antiviral CD8^+^ T cell function may occur due to the repetitive and sporadic HSV-1 latency and reactivation cycles, repetitive events that may take place in latently-infected TG of SYMP individuals^29–33^. Thus, even in the presence of a higher frequency of CD8^+^ T cells in the TG of seropositive individuals, HSV-1 can still manage to reactivate, depending on the function/exhaustion of TG-resident CD8^+^ T cells^3,6,34^. The molecular, phenotypic, and functional characteristics of antiviral CD8^+^ T cells associated with protective vs. pathogenic HSV-1 infections remain to be fully elucidated.

In the present study, we performed bulk and scRNASeq transcriptomic, phenotypic and functional analyses of blood and tissue-resident CD8^+^ T cells, specific to multiple and same HLA-A*0201-restricted HSV-1 epitopes from HSV-1 infected symptomatic and asymptomatic patients and from our HLA-A*0201 transgenic rabbit (HLA Tg rabbit) model of recurrent ocular herpes^18,28,31,35^. The identified molecular, phenotypic, and functional signatures of antiviral CD8^+^ T cells, sharing the same HSV-1 epitope-specificities, were confirmed in HSV-1-infected symptomatic and asymptomatic HLA Tg rabbits. Our findings reveal that higher frequencies of multifunctional memory CD8^+^ T_EM_ and T_RM_ cells, with an upregulation of the T cell activation, and T cell attracting chemokine/cytokine signaling pathways, are associated with asymptomatic herpes simplex infection. Moreover, blockade of immune checkpoints restored the functional CD8^+^ T cells and reduced virus reactivation from infected TG, and this was associated with a significant weakening of recurrent ocular herpes infection and disease in HLA transgenic rabbits. The findings from this study pave the way for T-cell-based immunotherapy to combat recurrent ocular herpes in humans.

## Results

### Major T cell exhaustion pathways are up-regulated in HSV-specific CD8^+^ T cells from symptomatic HSV-1 patients

**Figure 1:**
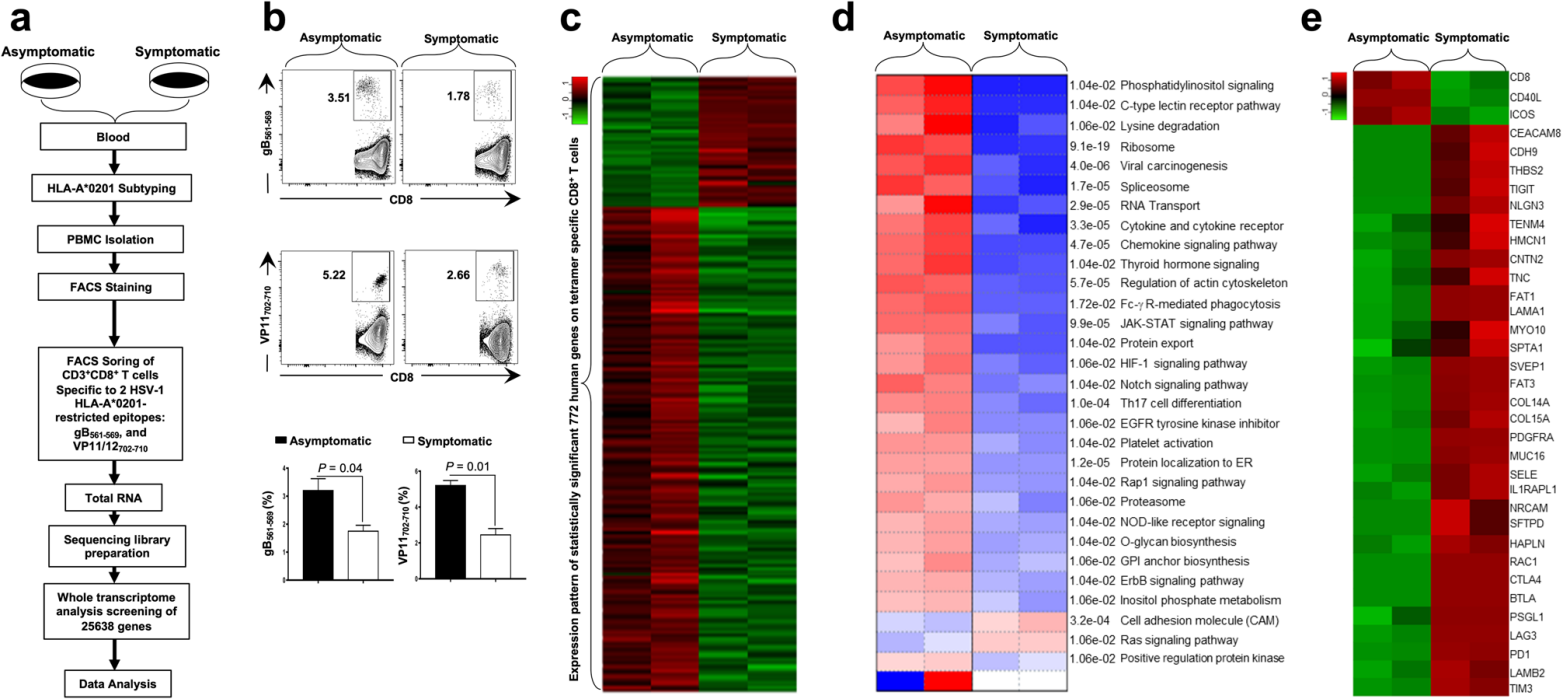
Differential gene expression in HSV-specific CD8^+^ T cells from HSV-1 infected symptomatic vs. asymptomatic individuals. (**a**) Experimental design and validation of differentially expressed genes in CD8^+^ T cells sharing the same HSV-1 epitope-specificities, from SYMP and ASYMP individuals. CD8^+^ T cells specific to HLA-A*0201-restricted HSV-1 gB_561–567_ and VP11/12_702–710_ epitopes were sorted from HLA-A*0201-positive SYMP and ASYMP individuals, using specific tetramers. Total RNA was extracted from each clone of epitope-specific CD8^+^ T cells, and whole transcriptome analysis was performed using bulk RNA sequencing to determine the levels of expression of 25,638 genes. (**b**) Frequencies of CD8^+^ T cells specific to HLA-A*0201-restricted HSV-1 gB_561–567_ and VP11/12_702–710_ epitopes detected by FACS in SYMP vs. ASYMP individuals. (**c**) Heatmap is showing 772 differentially expressed genes among SYMP and ASYMP individuals. (**d**) Heatmap showing statistically significant pathways that are affected in HSV-specific CD8^+^ T cells from SYMP vs. SYMP individuals. Parametric Gene Set Enrichment Analysis (PSGEA) method was applied based on data curated in Gene Ontology and KEGG. Pathway significance cut-off with a false discovery date (FDR) ≥ 0.2 was applied. (**e**) Bulk RNA heatmap comparing differentially expressed CAM pathway associated T cell co-stimulatory and T cell exhaustion genes in HSV-specific CD8^+^ T cells from SYMP vs. SYMP individuals.

Blood-derived CD8^+^ T cells, specific to two HLA-A*0201-restricted HSV-1 epitopes selected from the membrane glycoprotein B (gB_561–569_), and tegument protein VP11/12 (VP11–12_702–710_), were sorted (Supplementary Fig. S2) by fluorescence-activated cell sorting (FACS) from: (1) HLA-A*0201-positive SYMP individuals that exhibit frequent and severe recurrent ocular herpetic disease (*n* = 2); and (2) HLA-A*0201-positive ASYMP individuals that never have any recurrent herpetic disease despite being seropositive (*n* = 2) (Fig. 1a). Significantly higher frequencies of CD8^+^ T cells specific to HLA-A*0201-restricted HSV-1 epitopes gB_561–569_ (3.5% vs. 1.9%, *P* = 0.04) and VP11–12_702–710_ (5.4% vs. 2.7%, *P* = 0.01) were detected in ASYMP individuals compared to SYMP individuals (Fig. 1b).

Total RNA samples were isolated from sorted HSV-specific CD8^+^ T cells and subsequently processed for bulk RNA sequencing to screen the whole human transcriptome comprising 25,638 genes. Of this pool of genes, 20,126 genes were found with a minimum count per million (CPM) value ≥ 0.5. Our gene expression analysis revealed 772 genes to be statistically significant (FDR ≥ 0.1 and fold change ≥ 2) and differentially expressed among CD8^+^ T cells that share the same HSV-1 epitope-specificities, from SYMP vs. ASYMP groups (Fig. 1c). Of these genes, 583 genes were up-regulated, and 189 genes were down-regulated in HSV-specific CD8^+^ T cells from ASYMP individuals compared to SYMP patients (Supplementary Fig. S1a, upper panel). Correlation matrix and Pearson’s correlation coefficient confirmed a high degree of relatedness in the pattern of gene expression between SYMP and ASYMP individuals (Supplementary Fig. S1a, middle panel). The Volcano plot showed significant log2 fold changes and -log10 (FDR) of each of the differentially expressed genes in HSV-specific CD8^+^ T cells from ASYMP vs. SYMP patients (Supplementary Fig. S1a, lower panel). Further pathway enrichment analysis of the 772 differentially expressed genes between SYMP vs. ASYMP groups revealed a significant up-regulation of the cell adhesion molecule (CAM) pathway comprising major T cell exhaustion genes in HSV-specific CD8^+^ T cells from the SYMP group (*P* = 3.2e−04) (Fig. 1d). A bulk RNA sequencing specific heatmap confirmed the significant upregulation of CAM pathway-specific gene expression (i.e., *CEACAM8*, *NRCAM*, *LAMA1*, *SELE*, and *NLGN3*) and T cell exhaustion genes (i.e., *PD-1*, *LAG-3*, *PSGL-1*, *CTLA-4*, *TIM3*, and *TIGIT*) in HSV-specific CD8^+^ T cells from the SYMP patients compared to HSV-specific CD8^+^ T cells from the ASYMP individuals (Fig. 1e). These results indicate that an upregulation of CAM pathway associated genes along with major T cell exhaustion molecules in HSV-specific CD8^+^ T cells from SYMP patients is associated with frequent and severe recurrent ocular herpetic disease.

Due to the ethical and practical limitations in obtaining human TG samples from HSV-1-infected, SYMP and ASYMP individuals, the remainder of this study utilized our established HLA-A*0201 transgenic rabbit (HLA Tg rabbits) model of ocular herpes, which develops spontaneous virus reactivation, virus shedding in tears, and symptomatic recurrent ocular herpetic disease, as occurs in humans.

### Blockade of PD-1 and LAG-3 immune checkpoint reduces recurrent ocular herpes infection and disease in latently infected symptomatic HLA transgenic rabbits

Since up-regulation of genes for the *PD-1* and *LAG-3* exhaustion pathways in HSV-specific CD8^+^ T cells is associated with symptomatic herpes in humans, it was of interest to determine whether the blockade of PD-1 and LAG-3 immune checkpoint pathways would reduce virus reactivation and ease symptomatic recurrent ocular herpes.

**Figure 2:**
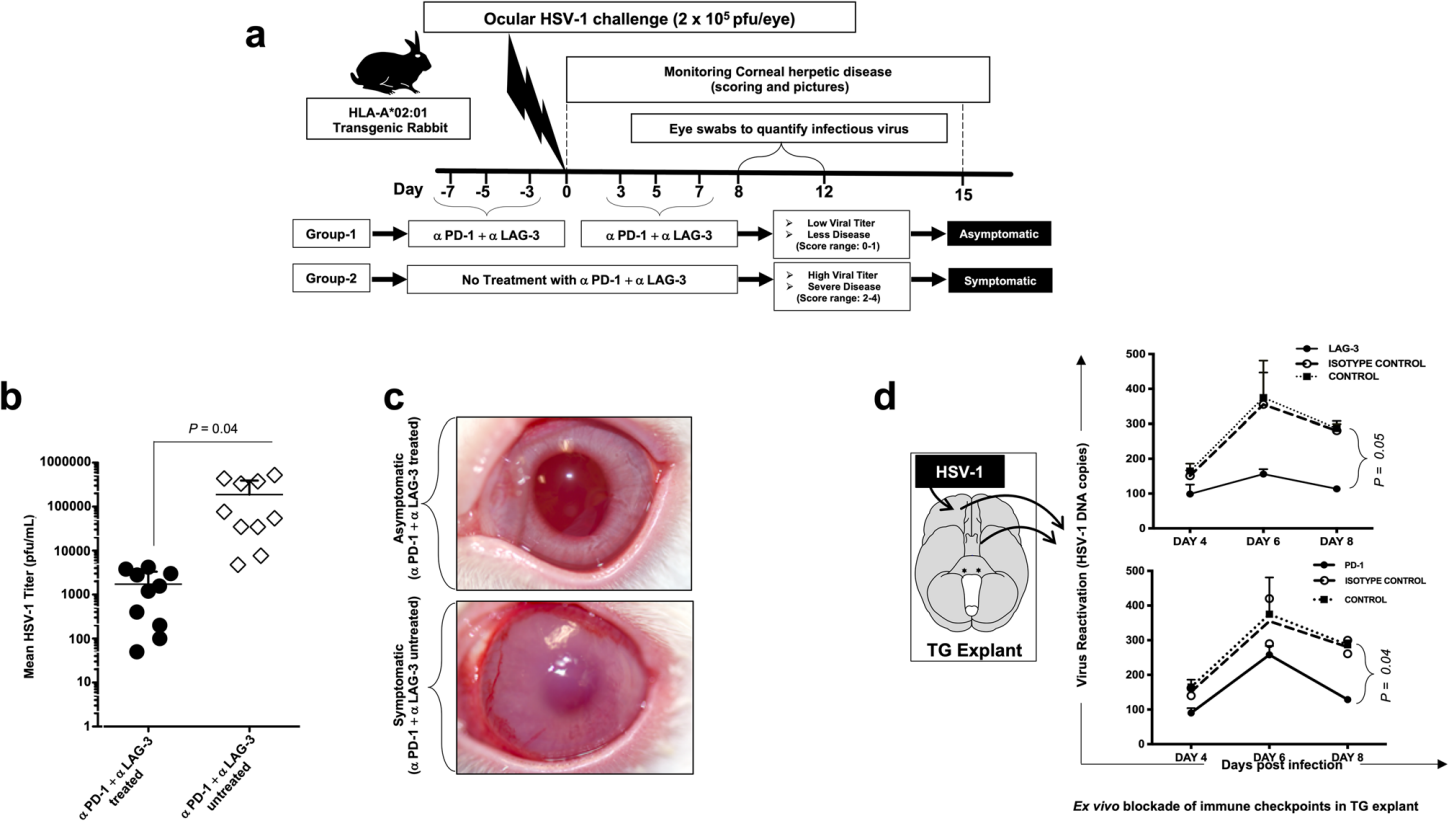
Effect of immune checkpoint-blockade on recurrent ocular herpes infection and disease in HSV-1 infected HLA Tg rabbits. (**a**) Schematic representation of HSV-1 ocular infection, α-PD-1/α-LAG-3 mAbs treatment, immunological, virological, and disease analyses in HLA Tg rabbits ocularly infected with HSV-1. HLA-Tg rabbits from Group-1 were ocularly infected with HSV-1 and treated with α-PD-1/α-LAG-3 mAbs whereas HLA-Tg rabbits placed in Group-2 were also ocularly infected with HSV-1 but not treated with α-PD-1/α-LAG-3 mAbs. Based on the severity and frequency of recurrent ocular herpetic disease, the rabbits were scored between 0 and 4 and subsequently categorized into SYMP and ASYMP groups. (**b**) HSV-1 viral titers in the eyes of α-PD-1/α-LAG-3 treated and α-PD-1/α-LAG-3 untreated HLA Tg rabbits. Infectious virus particles were quantified from eye swabs of α-PD-1/α-LAG-3 treated and α-PD-1/α-LAG-3 untreated HLA Tg rabbits. (**c**) Representative images showing recurrent ocular herpetic disease in HSV-1 infected SYMP HLA Tg rabbits. (**d**) Trigeminal ganglia were extracted from HSV-1 infected non-treated HLA Tg rabbits on day 15 post-infection. The effect of α-PD-1 or α-LAG-3 mAbs treatment on virus reactivation, ex vivo from TG explants, was assessed along with that of isotype mAb control.

As illustrated in Fig. 2a, HLA Tg rabbits (*n* = 20) were infected with 2 × 10^5^ pfu of HSV-1 (McKrae Strain). Half of the rabbits (Group-1; *n* = 10) were treated with a combination of blocking α-PD-1 and α-LAG-3 mAbs, injected intravenously (i.v.) on days -3, -5, -7 before infection and then on days 3, 5, and 7 post-infection (p.i.) at 200 μg/dose. The other half of the rabbits (Group-2; *n* = 10) received saline injections (untreated controls). Eye swabs were collected daily for 5 days p.i., and recurrent ocular herpetic disease was monitored 15 days p.i. Rabbits were subsequently segregated into (1) asymptomatic (ASYMP) rabbits, with no apparent recurrent ocular herpetic disease, and (2) symptomatic (SYMP) rabbits with higher rates and severe recurrent ocular herpetic disease. The detailed characteristics of the SYMP and ASYMP HLA Tg rabbits used are described in “Methods”. The α-PD-1/α-LAG-3 mAbs-treated group had the most ASYMP animals, with significantly less virus titers in the eyes (*P* < 0.04, Fig. 2b) and no apparent recurrent corneal herpetic disease (Fig. 2c). In contrast, the untreated group had the most SYMP animals, which showed a significantly higher level of virus replication in eyes associated with severe recurrent ocular herpetic disease (*P* < 0.04, Fig. 2b,c).

It was next determined whether the observed reduction of virus replication in the eyes of ASYMP HLA Tg rabbits treated with PD-1 and LAG-3 immune checkpoint blockade was associated with a reduction in virus reactivation locally in the TG, the site of latent HSV-1 infection/reactivation cycles. The viral load during ex-vivo TG reactivation is often determined using RT-PCR as a more sensitive alternative to plaque assay^34,36,37^. The excised latently infected TG from the same rabbit was digested and equally distributed in control, and the mAb treated wells, so the possibility of variation in DNA copy numbers due to variation in the level of latency does not arise. The amount of reactivated virus detected ex vivo in treated and untreated HSV-1 infected TG explants was determined daily for 8 days post-treatment. As shown in Fig. 2d, the HSV-1 infected TG explants incubated with α-PD-1, or α-LAG-3 mAbs showed a significant decrease of the reactivated virus on day 8 following the blockade (*P* < 0.05). The blockade of the LAG-3 pathway (*P* = 0.04) appeared slightly better in reducing virus reactivation from TG compared to blockade of the PD-1 pathway (*P* < 0.05).

Altogether, these results demonstrate that blockade of PD-1 and/or LAG-3 immune checkpoints reduced recurrent ocular herpes infection and disease in vivo in latently infected HLA Tg rabbits. The observed reduction of virus replication and recurrent ocular herpetic disease in PD-1 and LAG-3 treated HLA Tg rabbits was associated with a significant reduction in virus reactivation locally in the HSV-1 infected TG.

Since the HLA Tg rabbit model develops human-like CD8^+^ T cell responses to HLA-A*0201 restricted epitopes^3,21,38^, it offers the possibility to determine the phenotype, function, and transcriptome of TG-resident HLA-A*0201 restricted HSV-1 epitopes-specific CD8^+^ T cells associated with symptomatic vs. asymptomatic recurrent ocular herpes.

### Single-cell RNA sequencing revealed CD8^+^ T cells and monocytes as the most significant CD45^+^ leukocytes infiltrating trigeminal ganglia of "protected" asymptomatic HLA Tg rabbits

**Figure 3:**
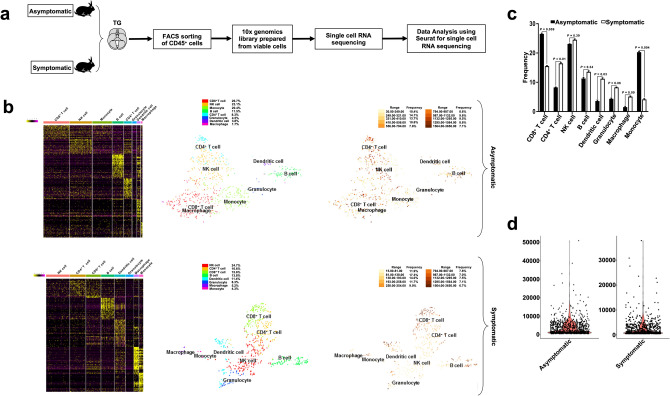
Single-cell RNA sequencing of trigeminal ganglia-resident CD45^+^ leukocytes from HSV-1 infected symptomatic vs. asymptomatic HLA Tg rabbits*.* (**a**) Illustration of the experimental design and validation of differentially expressed genes in CD45^+^ leukocytes sorted on day 15 p.i. from the trigeminal ganglia (TG) of SYMP and ASYMP HLA Tg rabbits. (**b**) Heatmap expression of the most significant 140 differentially expressed genes among eight different clusters detected in TG-resident CD45^+^ leukocytes from HSV-1 infected SYMP and ASYMP HLA Tg rabbits (top two heatmap panels). Each cluster represents an individual immune cell population, determined on the basis of specific molecular markers: CD8^+^ T cells (CD8A), CD4^+^ T cells (CD4), NK cells (NKG7), B cells (CD19), macrophages (CD68), monocytes (CD14), granulocytes (FUT4) and dendritic cells (CD1c). The t-SNE dimensionality reduction, applied to single-cell RNA sequencing data revealed eight distinct clusters of immune cell populations among CD45^+^ leukocytes for the TG of HSV-1 infected ASYMP HLA Tg rabbits (middle panels). The total number of differentially expressed genes within each immune cell clusters (nCount) (lower panels). (**c**) Average frequencies of different immune cell populations detected within TG-resident CD45^+^ leukocytes of SYMP and ASYMP HLA Tg rabbits. (**d**) Volcano plot illustrates the total copy number reads observed for all the genes within one single cell (nFeature).

TG-derived CD45^+^ leukocytes were sorted by fluorescence-activated cell sorting (FACS) from (1) SYMP HLA Tg rabbits (*n* = 4); and (2) ASYMP HLA Tg rabbits (*n* = 4), as illustrated in Figs. 2a and 3a and subsequently processed for single-cell RNA sequencing (scRNASeq) using the 10 × Genomics platform. Eight cell populations were observed among the sorted CD45^+^ leukocytes from TG of ASYMP and SYMP groups: CD4^+^ T cells, CD8^+^ T cells, NK cells, B cells, macrophages, monocytes, granulocytes, and dendritic cells (Fig. 3b).

CD8^+^ T cells (defined by *CD8A* gene) and monocytes (defined by *CD14* gene) represented the most frequent CD45^+^ leukocyte populations in the TG of "protected" ASYMP HLA Tg rabbits compared to TG of "non-protected" SYMP HLA Tg rabbits. We detected a total of 198 (26.7%) CD8^+^ T cells per TG in ASYMP HLA Tg rabbits, while only 116 (15.6%) CD8^+^ T cells were found in the TG of SYMP HLA Tg rabbits (Fig. 3c). After CD8^+^ T cells, the next most significant cell population detected in the TG of ASYMP HLA Tg rabbits were the monocytes at a frequency of 20.4% of the total cell population in comparison to only 4.3% among the SYMP HLA Tg rabbits (Fig. 3c). A relatively higher mRNA copy number was also found in the TG of ASYMP HLA Tg rabbits (Fig. 3d).

These results indicate that: (1) anti-PD-1 and anti-LAG-3 blockade induced compartmental remodeling of TG-infiltrating immune cells of HSV-infected HLA Tg rabbits; and (2) expansion of CD8^+^ T cells and monocytes in the TG of HSV-1 infected asymptomatic HLA Tg rabbits is associated with reduced virus reactivation and less recurrent ocular herpetic disease.

### Up-regulation of major T cell exhaustion pathways confirmed by bulk RNA sequencing in TG-resident HSV-specific CD8^+^ T cells from symptomatic HLA Tg rabbits

**Figure 4:**
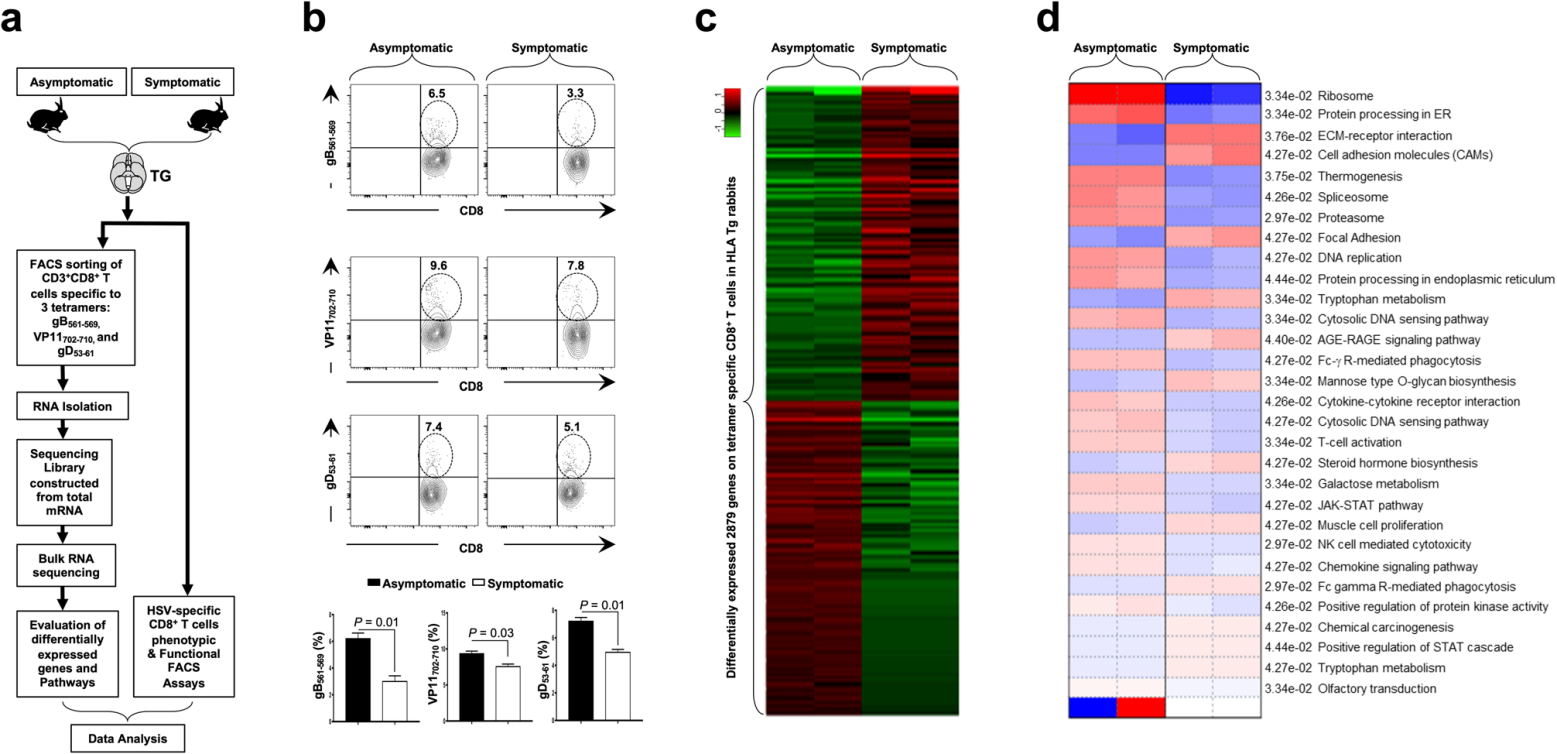
Differential gene expression in HSV-specific CD8^+^ T cells from trigeminal ganglia of HSV-1 infected symptomatic vs. asymptomatic HLA Tg rabbits*.* (**a**) Experimental design and validation of differentially expressed genes in CD8^+^ T cells sharing the same HSV-1 epitope-specificities, from SYMP and ASYMP HLA Tg rabbits. CD8^+^ T cells specific to HLA-A*0201-restricted HSV-1 gB_561–567_, VP11/12_702–710,_ and gD_53–61_ epitopes were sorted from TG of HLA-A*0201-positive SYMP and ASYMP HLA Tg rabbits, using specific tetramers. Total RNA was extracted from each clone of epitope-specific CD8^+^ T cells, and whole transcriptome analysis was performed using bulk RNA sequencing to determine the levels of expression of 23,669 rabbit genes (OryCun2.0 (GCA_000003625.1). (**b**) Frequencies of CD8^+^ T cells specific to HLA-A*0201-restricted HSV-1 gB_561–567_, VP11/12_702–710,_ and gD_53–61_ epitopes detected by FACS in TG of HLA-Tg rabbits. (**c**) The heatmap is showing the most significant 2,879 differentially expressed genes among SYMP and ASYMP HLA Tg rabbits. Genes with minimum count per million (CPM) ≥ 0.5 were used for obtaining the transformed counts data for clustering using regularized log (rlog). (**d**) Bulk RNA heatmap shows the pathways that are different among ASYMP and SYMP HLA Tg rabbits. Genes differentially expressed in both single-cell RNA sequencing and bulk RNA sequencing were considered for pathway analyses. Parametric gene set enrichment analysis (PSGEA) method based on data curated in Gene Ontology and KEGG was applied. Pathway significance cut-off with a false discovery date (FDR) ≥ 0.2 was applied.

TG-derived CD8^+^ T cells, specific to three HLA-A*0201-restricted HSV-1 epitopes selected from the glycoprotein B (gB_561–569_), the glycoprotein D (gD_53–61_), and the tegument protein VP11/12 (VP11–12_702–710_), were enriched by fluorescence-activated cell sorting (FACS) from: (1) SYMP HLA Tg rabbits (*n* = 2); and (2) ASYMP HLA Tg rabbits (*n* = 2), as illustrated in Fig. 4a. Higher frequencies of CD8^+^ T cells specific to HLA-A*0201-restricted HSV-1 epitopes gB_561–569_ (6.5% vs. 3.3%, *P* = 0.01), VP11–12_702–710_ (9.6% vs. 7.8%, *P* = 0.03), and gD_53–61_ (7.4% vs. 5.1%, *P* = 0.01) were detected in HSV-1 infected and protected ASYMP HLA Tg rabbits compared to non-protected SYMP HLA Tg rabbits (Fig. 4b).

Total RNA was isolated from sorted CD8^+^ T cells and subsequently processed for bulk RNA sequencing to screen the whole rabbit transcriptome. Our gene expression analysis revealed 12,689 genes among CD8^+^ T cells, with the same HSV-1 epitope-specificities, from ASYMP vs. SYMP HLA Tg rabbits with a minimum count per million (CPM) value ≥ 0.5. Out of these genes, 2,879 genes were found to be statistically significant and differentially expressed between ASYMP and SYMP HSV-specific CD8^+^ T cells using stringent criteria (FDR-adjusted *P* < 0.05, fold change ≥ 2, paired moderated two-tailed *t* test) (Fig. 4c). Further, among the 2,879 genes, we found 1,605 genes were up-regulated, while 1,274 genes were down-regulated in HSV-specific CD8^+^ T cells from TG of ASYMP HLA Tg rabbits (Supplementary Fig. S1b, upper panel). Correlation matrix and Pearson's coefficient confirmed a high degree of relatedness amongst the SYMP and ASYMP HLA Tg rabbit groups (Supplementary Fig. S1b, middle panel). The Volcano plot showed significant log2 fold changes and − log10 (*P* value) of each of the differentially expressed genes in CD8^+^ T cells from SYMP vs. CD8^+^ T cells, that shared the same HSV-1 epitopes, from SYMP HLA Tg rabbits (Supplementary Fig. S1b, lower panels).

The pathway enrichment analysis carried out on the basis of differentially expressed genes among scRNA-Seq, and bulk RNA-Seq demonstrated downregulation of prominent T cell exhaustion molecules present in the cell adhesion molecules (CAMs) pathway (*P* = 0.04) (Accession no: ocu04514) among the ASYMP group (Fig. 4d). The CAMs pathway is comprised of *PD-1-*, *LAG-3-*, *TIM3-*, *TIGIT-*, *CTLA4-*, *PSGL-1* genes. In contrast, the T cell activation pathway (*P* = 0.03) (Accession no: GO: 0042110), the chemokine-chemokine receptor signaling pathway (*P* = 0.04) (Accession no: ocu04062), and the cytokine-cytokine receptor interaction (*P* = 0.04) (Accession no: ocu04060) were all up-regulated in TG-resident HSV-specific CD8^+^ T cells from ASYMP HLA Tg rabbits (Fig. 4d).

These results indicate that similar to SYMP patients above, in SYMP HLA Tg rabbits, there was an up-regulation of T cell exhaustion pathways in HSV-specific CD8^+^ T cells associated with frequent and severe recurrent ocular herpetic disease.

### High frequencies of HSV-specific memory CD8^+^ T_EM_ and CD8^+^ T_RM_ cell subsets, with upregulated T-cell activation pathways, detected in TG of asymptomatic HLA Tg rabbits

We next compared the frequencies of the three major subsets of memory CD8^+^ T cells that share the same HSV-1 epitope-specificities and are present in the TG of ASYMP vs. SYMP HLA Tg rabbits. As illustrated in Fig. 2a above, HLA Tg rabbits (*n* = 8) were first infected with 2 × 10^5^ pfu of HSV-1 (McKrae Strain).

**Figure 5:**
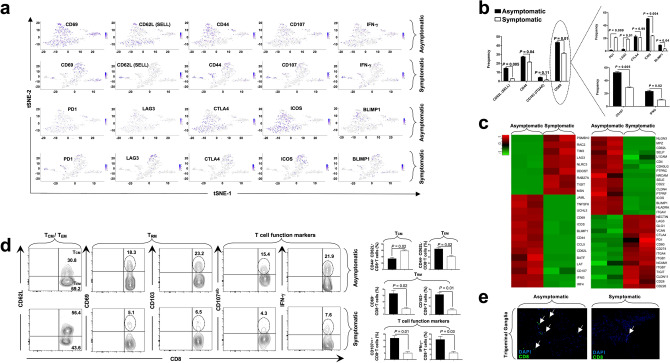
Activation and exhaustion genes differentially expressed in trigeminal ganglia-resident HSV-specific CD8^+^ T cells from HSV-1 infected symptomatic vs. asymptomatic HLA Tg rabbits*.* (**a**) Expression of T cell activation genes (*CD69*, *CD62L*, *CD44*, *CD107*, and *IFN-γ*) detected by single-cell RNA sequencing from SYMP vs. ASYMP HLA Tg rabbits is represented through t-SNE plots (top panels). Expression of T cell exhaustion genes (*PD-1*, *LAG-3*, *CTLA4*, *ICOS*, and *BLIMP1*) detected by single-cell RNA sequencing from SYMP vs. ASYMP HLA Tg rabbits is represented through t-SNE plots (lower panels). (**b**) Average frequencies of specific genes representing memory CD8^+^ T_CM_, CD8^+^ T_EM_, and CD8^+^ T_RM_ cell subsets from TG of SYMP vs. ASYMP HLA Tg rabbits (left panel). Average frequencies of CD8^+^ T_RM_ cells expressing various exhaustion genes in HSV-1 infected TG of in SYMP vs. ASYMP HLA Tg rabbits (top right panel). Average frequencies of functional CD107^a/b+^IFN-*γ* ^+^CD8^+^ T_RM_ cells in HSV-1 infected TG of in SYMP vs. ASYMP HLA Tg rabbits (lower right panel). (**c**) Bulk RNA sequencing showing expression of T-cell activation (left panel) and T-cell exhaustion genes (right panel) in HSV-specific T_RM_ cells from SYMP vs. ASYMP HLA Tg rabbits. (**d**) Frequencies of memory CD8^+^ T_CM_, CD8^+^ T_EM_, and CD8^+^ T_RM_ cell subsets detected by FACS in HSV-1 infected TG of SYMP vs. ASYMP HLA Tg rabbits. (**e**) Fluorescence microscopy images showing infiltration of CD8^+^ T cells in HSV-1 infected TG from SYMP vs. ASYMP HLA Tg rabbits. TG sections were co-stained using DAPI and mAb specific to rabbit CD8^+^ T cells (magnification, × 20). Blue, DAPI: DNA, green: CD8^+^ T cells.

As shown in Fig. 5a, several genes associated with T-cell activation pathway were upregulated in HSV-specific CD8^+^ T cells from TG of “protected” ASYMP HLA Tg rabbits, as compared to HSV-specific CD8^+^ T cells from TG of “non-protected” SYMP HLA Tg rabbits (*P* < 0.05). Prominent genes that were found to be significantly upregulated among ASYMP group include *CD69* (*P* = 0.03, logFC = 2.72), *CD62L* (*P* = 0.004, logFC = 6.48), *CD44* (*P* = 1.52E−06, logFC = 2.79), *CD107* (*P* = 7.04E–05, logFC = 2.14), and *IFN-γ* (*P* = 0.01, logFC = 2.03) (Fig. 5a; Supplementary Table S1).

In the cell cluster representing 198 CD8^+^ T cells; 86 cells (43.4%) expressed *CD69* gene, 28 cells (14.1%) expressed *CD62L* gene, 54 cells (27.2%) expressed *CD44* gene, and 8 cells (4.5%) expressed *CD107* gene (Fig. 5b). Notably, the *IFN-γ* and *CD107* genes were expressed in 22.09% (*P* = 0.005) and 53.4% (*P* = 0.02) of CD69^+^CD8^+^ T_RM_ cells in TG of ASYMP HLA Tg rabbits (Fig. 5b, left lower panel). In contrast, only ~ 20% of CD69^+^CD8^+^ T_RM_ cells from TG of “non-protected” SYMP HLA Tg rabbits expressed genes for the PD-1 and LAG-3 exhaustion pathway compared to less than 5% of CD69^+^CD8^+^ T_RM_ cells from TG of “protected” SYMP HLA Tg rabbits that expressed PD-1 and LAG-3 exhaustion genes (*P* ≤ 0.01) (Fig. 5b, left upper panel).

The CAMs pathway-specific genes were significantly down-regulated in TG-resident CD103^+^CD69^+^CD8^+^ T_RM_ cells from ASYMP HLA Tg rabbits compared to TG-resident CD103^+^CD69^+^CD8^+^ T_RM_ cells, with same epitopes-specificities, from SYMP HLA Tg rabbits. Major T cell exhaustion molecules like *PD-1* (*P* = 0.005, logFC = − 2.02), *LAG-3* (*P* = 0.04, logFC = − 4.15), *CTLA4* (*P* = 0.02, logFC = − 1.78), were found to be downregulated among ASYMP HLA Tg rabbits in the CD8^+^ T_RM_ cells as evidenced from the single cell RNA sequencing results (Fig. 5a; Supplementary Table S2). Whereas, Inducible T-cell Co-Stimulator (*ICOS*) gene (*P* = 0.04, logFC = 4.03), and *PRDM1* (*BLIMP1*) (*P* = 0.01, logFC = 3.1) known to be associated with T cell activation and differentiation were observed with a higher expression in the TG resident T_RM_ cells among ASYMP HLA Tg rabbits (Fig. 5a; Supplementary Table S2).

*PD*-1 and *LAG-3* genes were expressed in 21.6% and 18.9% of TG-resident CD103^+^CD69^+^CD8^+^ T_RM_ cells in the SYMP HLA Tg rabbits compared to only 1.16% and 2.3% of TG-resident CD103^+^CD69^+^CD8^+^ T_RM_ cells in the ASYMP HLA Tg rabbits (Fig. 5b).

When differential gene expression analysis was carried out between ASYMP vs. SYMP groups using bulk RNA sequencing, *TIGIT* (*P* = 0.05, logFC = − 2.70), *CTLA4* (*P* = 0.01, logFC = − 1.04), *PD-1* (*P* = 1.01E−06, logFC = − 5.58) genes were found to be significantly down-regulated along with other T cell exhaustion molecules (Fig. 5c, right panel; Supplementary Table S2). The bulk RNA sequencing-based on differential gene expression (DGE) analysis performed on TG-resident memory CD103^+^CD69^+^CD8^+^ T_RM_ cells, with the same HSV-1 epitope-specificities, confirmed a heightened expression level of several genes associated with the T cell activation pathway in "protected" ASYMP HLA Tg rabbits (Fig. 5c). In contrast, significantly higher expression levels of several genes associated with the T cell exhaustion were confirmed by bulk RNA sequencing in "non-protected" SYMP HLA Tg rabbits (Fig. 5c). Importantly, the single-cell RNA sequencing-based transcriptome of CD45^+^ leukocytes clearly showed increased frequencies of memory CD8^+^ TRM and CD8^+^ TEM cell subsets in the TG of "protected" ASYMP HLA Tg rabbits.

Moreover, these genomic results were supported by FACS-based immunophenotyping, which confirmed higher frequencies of the memory CD8^+^ T_RM_ cell subset (ASYMP = 69.2% vs SYMP = 43.6%, *P* = 0.02) and memory CD8^+^ T_EM_ cell subset expressing adhesion molecules, CD69 (ASYMP = 18.3% vs SYMP = 5.1%, *P* = 0.02) and CD103 (ASYMP = 23.2% vs SYMP = 6.5%; *P* = 0.01) in the TG of “protected” ASYMP HLA Tg rabbits (Fig. 5d). More-so, an increased infiltration of CD8^+^ T cells in the TG of ASYMP rabbits was visualized by immunostaining (Fig. 5e).

Interestingly, Fig. 5d shows upregulation of *CD62L* protein expression at the surface of the HSV-specific CD8^+^ T-cells from SYMP compared to ASYMP individuals, whereas the single-cell and bulk RNAseq data panels on Fig. 5a–c show downregulation of the *CD62L* mRNA expression in SYMP subjects. While the protein expression for the other markers (*CD69*, *CD44*, *CD103*) follows their mRNA expression, this does not seem to be the case with *CD62L*. This may be due to post-transcriptional/translational modification/regulation. Thus, it appears that the expression of *CD62L* on the surface on the T-cell (at the protein level) is regulated not only at the transcriptional level but also at a post-transcriptional level. In previous reports, it has been shown that the loss of *CD62L* by the activated T-cells can be regulated by the cleavage of *CD62L* from the cell membrane at K283–S284 by a disintegrin and metalloprotease ADAM17 in a process called CD62L shedding^39–42^.

### Heightened expression of genes for T cell attracting chemokines/receptors and T cell maintaining cytokines/receptors detected in TG-resident CD8^+^ T_RM_ cells from asymptomatic HLA Tg rabbits

The higher frequencies of HSV-specific CD8^+^ T_RM_ cell subset observed in the TG of "protected" ASYMP HLA Tg rabbits prompted us to further compare in CD8^+^ T cells TG from SYMP vs. ASYMP HLA Tg rabbits the differential gene expression profile of genes for cytokines, chemokines and their receptors that are involved in T cell homing and T cell maintaining.

**Figure 6:**
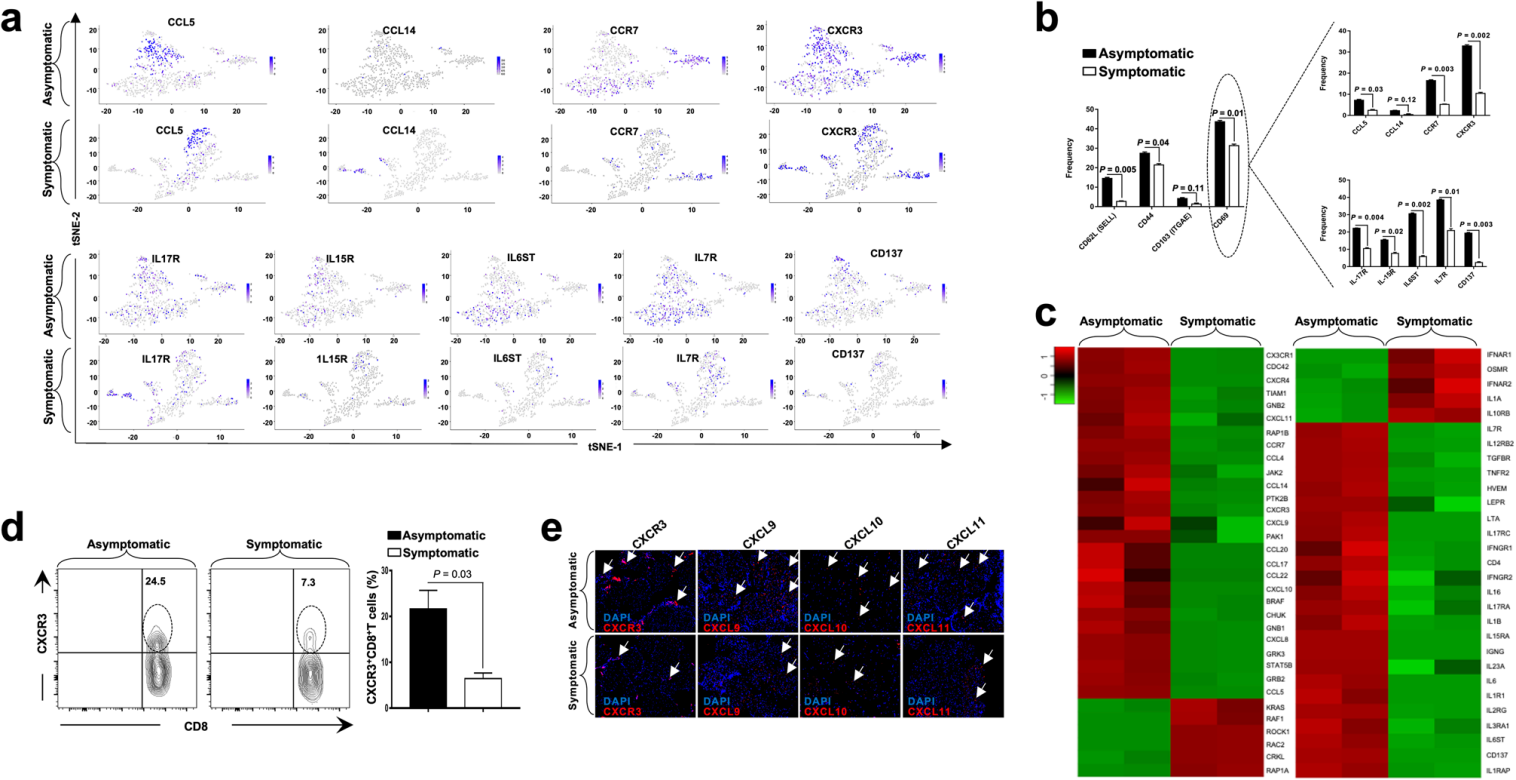
Genes of cytokines/chemokines and receptors differentially expressed in trigeminal ganglia-resident HSV-specific CD8^+^ T cells from HSV-1 infected symptomatic vs. asymptomatic HLA Tg rabbits*.* (**a**) Expression of genes of chemokines and chemokine receptors detected by single-cell RNA sequencing from TG-resident HSV-specific CD8^+^ T cells from SYMP vs. ASYMP HLA Tg rabbits is represented through t-SNE plots (top panels). Expression of genes of cytokines and cytokine receptors detected by single-cell RNA sequencing from TG-resident HSV-specific CD8^+^ T cells from SYMP vs. ASYMP HLA Tg rabbits is represented through t-SNE plots (lower panels). (**b**) Average frequencies of CD8^+^ T_RM_ cells expressing genes of cytokines/chemokines and receptors in TG of HSV-1 infected SYMP vs. ASYMP HLA Tg rabbits. (**c**) Bulk RNA sequencing showing expression of genes of chemokines/chemokine receptors (left panel) and genes of cytokines/cytokine receptors (right panel) in HSV-specific T_RM_ cells from SYMP vs. ASYMP HLA Tg rabbits. (**d**) Representative (left panels) and average (right panels) frequencies of CCR3^+^CD8^+^ T_RM cells_ in TG of HSV-1 infected SYMP vs. ASYMP HLA Tg rabbits. (**e**) Fluorescence microscopy images showing infiltration of HSV-1 infected TG from SYMP vs. ASYMP HLA Tg rabbits by CD8^+^ T cells expressing T cell-attracting chemokines and receptors (i.e., CXCR3, CXCL9, CXCL10, and CXCL11) (magnification, × 20). Blue: DAPI (DNA stain); red: CXCR3 cells.

Using both the single cell and bulk RNA sequencing methods, we detected a significant up-regulation of several genes for T cell attracting chemokines and chemokine receptors in TG-resident HSV-specific CD103^+^CD69^+^CD8^+^ T_RM_ cells from ASYMP HLA Tg rabbits. The genes found to be significantly upregulated among ASYMP HLA Tg rabbits represented CC-, CXC-, and CX3C-subfamilies of chemokines including *CCL5* (*P* = 4.13E−26, logFC = 3.29), *CCL14* (*P* = 0.05, logFC = 3.02), *CCR7* (*P* = 7.52E−05, logFC = 5.98), and *CXCR3* (*P* = 0.02, logFC = 2.63). (Fig. 6a,c, left panel; Supplementary Table S3). *CCL5*, *CCR7*, and *CXCR3* genes were expressed in 6.9%, 16.2%, and 32.5% of CD8^+^ T_RM_ cells in the ASYMP group compared to 2.7%, 5.4%, and 10.8% of CD8^+^ T_RM_ cells, with the same epitope specificities, in the SYMP group (Fig. 6b, upper panel).

Single cell and bulk RNA sequencing results (Fig. 6a,c, right panel) also indicate that several genes associated with the cytokine–cytokine receptor interaction pathway that are known for maintaining T cells within tissues were up-regulated among the ASYMP group of HLA Tg rabbits: *IL-17R* (*P* = 0.008, logFC = 2.13), *IL-15R* (*P* = 0.01, logFC = 2.51), *IL6ST* (*P* = 0.04, logFC = 6.69), *IL7R* (*P* = 4.13E−07, logFC = 4.35), *CD137* (*P* = 2.17E−12, logFC = 2.35) to be upregulated in the CD8^+^ T_RM_ cells among the ASYMP group of HLA Tg rabbits (Fig. 6a; Supplementary Table S4). The expression of these cytokines/cytokine receptors were significantly increased among the CD8^+^ T_RM_ cell subset in ASYMP HLA Tg rabbits. The expression of genes *IL-17R*, *IL-15R*, *IL6ST*, *IL7R*, and *CD137* (*TNFRSF9*) was in 22.09% (*P* = 0.004), 15.1% (*P* = 0.02), 30.2% (*P* = 0.002), 38.3% (*P* = 0.01) and 19.7% (*P* = 0.003) of CD8^+^ T_RM_ cells, respectively (Fig. 6b).

Furthermore, we confirmed higher frequencies of CXCR3^+^CD8^+^ T_RM_ cells in TG of ASYMP HLA Tg rabbits compared to TG of SYMP HLA Tg rabbits (ASYMP = 24.5%, SYMP = 7.3%; *P* = 0.03) by FACS (Fig. 6d). Using differential gene expression analysis between the ASYMP vs. SYMP groups, the genes for T cell attracting-chemokines *CXCL9* (*P* = 0.003, logFC = 3.70), *CXCL10* (*P* = 0.001, logFC = 4.32), and *CXCL11* (*P* = 0.01, logFC = 2.84) genes were also found to be significantly up-regulated in TG-resident CD8^+^ T_RM_ cells from “protected” ASYMP HLA Tg rabbits compared to TG-resident CD8^+^ T_RM_ cells from “non-protected” SYMP HLA Tg rabbits (Fig. 6c). Similarly, increased infiltration of CXCR3^+^CD8^+^ T_RM_ cells in the TG of ASYMP rabbits was visualized by immunostaining (Fig. 6e).

**Figure 7:**
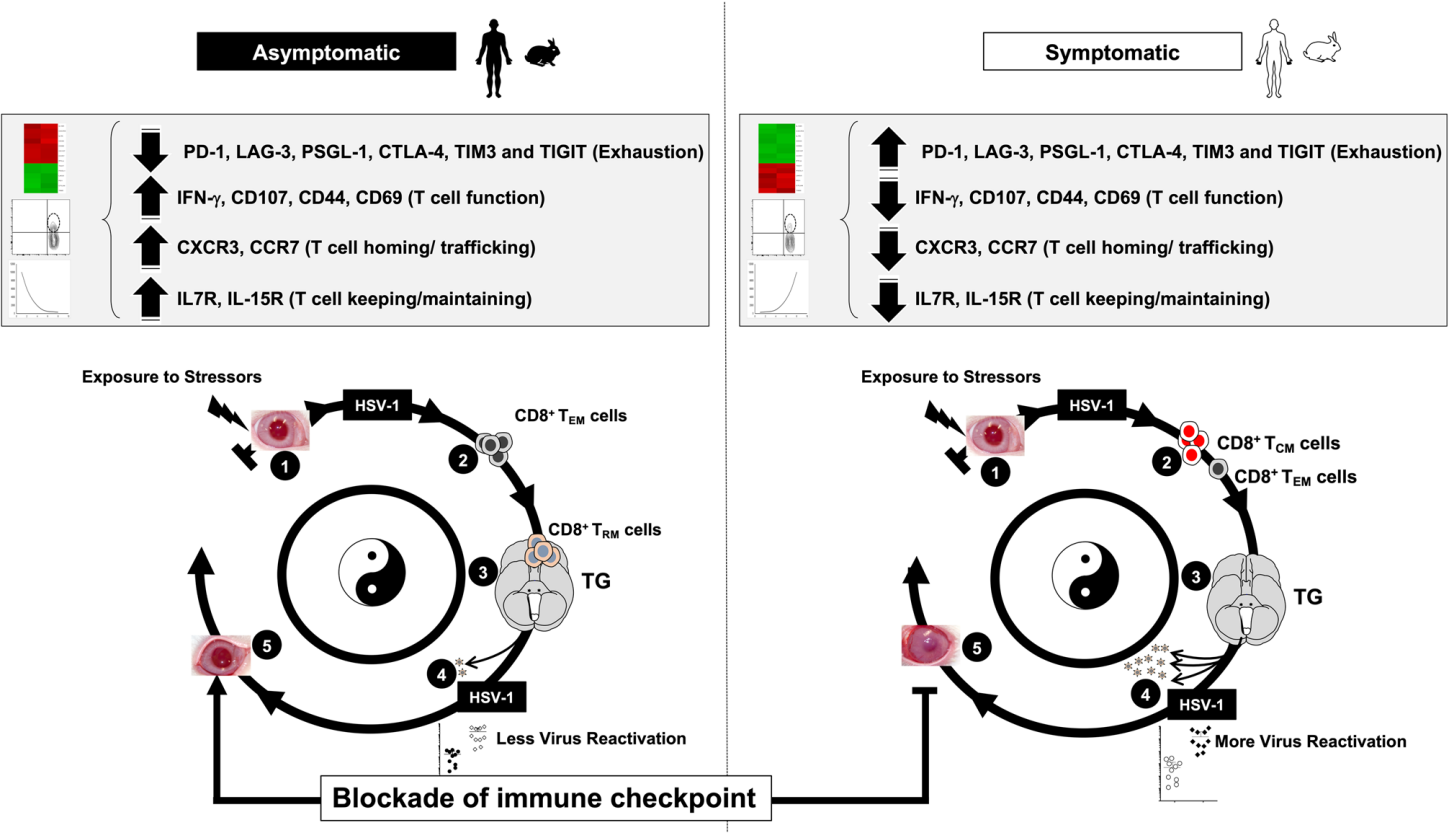
TG-resident HSV-specific memory CD8^+^ T_RM_ cells downregulate the T cell exhaustion associated pathway and confer protection from ocular herpes in HSV-1 infected asymptomatic humans and HLA transgenic rabbits. (**1**) Upon exposure to stressors, the HSV-1 enters into the cornea and travels through neurons to Trigeminal ganglia. (**2**) Following primary HSV-1 infection, the vast majority (up to 95%) of antiviral effector CD8^+^ T cells die, leaving behind only about 5% of CD8^+^ T cells destined to differentiate into a heterogeneous pool of memory CD8^+^ T cells. (**3**) The effector memory (T_EM_) and tissue-resident memory (T_RM_) CD8^+^ T-cell subsets are found mainly in the HSV-infected but "naturally protected" asymptomatic subjects, whereas the lymphoid organ-resident central memory (T_CM_) CD8^+^ T cell subsets are mainly present in non-protected Symptomatic subjects. (**4**) Reduced viral reactivation was observed among asymptomatic subjects possessing a higher frequency of CD8^+^ T_RM_ cells resulting in a less severe herpes disease. (**5**) The findings study suggests that by blocking immune checkpoints, there is a reduced expression of T cell exhaustion molecules (*PD-1*, *LAG-3*, *PSGL-1*, *CTLA-4*, *TIM3*, and *TIGIT*) and T cell exhaustion associated Cell Adhesion Molecule pathway and increased retention of CD8^+^ T_RM_ cell population in asymptomatic subjects. This memory CD8^+^ T cell population mediates recall responses and halts attempts of virus reactivation in the infected TG, thus accelerating viral clearance. More-so, reduced expression of T cell exhaustion pathway also gives rise to higher expression of genes associated with T cell function (*CD107*, *IFN-γ*), T cell homing (*CXCR3*, *CCR7*), and T-cell keeping (*IL7R*, *IL15R*). This helps in reducing the ocular herpes infection and recurrent herpetic disease.

Altogether, these results suggest that a heightened activation of T cell attracting chemokines/receptors and T cell keeping cytokines/receptors pathways may lead to increased infiltration/retention of protective antiviral CXCR3^+^CD8^+^ T_RM_ cells observed in the TG of "protected" ASYMP HLA Tg rabbits, as illustrated in Fig. 7.

## Discussion

We report significant differences in the phenotype, function, and molecular signatures of HSV-specific CD8^+^ T cells that are associated with symptomatic vs. asymptomatic recurrent ocular herpes infection in humans and HLA Tg rabbits. We were not able to detect tetramer-positive HSV-specific CD8^+^ T cells in the herpes seronegative control individuals. Due to the lack of HSV-specific CD8^+^ T cells, we were not able to include these cells in the RNA sequencing experiments as baseline control. Compared to multifunctional blood-derived CD8^+^ T_EM_ cells in ASYMP individuals, SYMP individuals displayed high frequencies of dysfunctional HSV-specific blood-derived effector memory CD8^+^ T_EM_ cells, expressing genes of major exhaustion pathways. Similarly, the trigeminal ganglia of "protected" ASYMP HLA Tg rabbits contain a high number of functional CD8^+^ T cells with phenotypic and functional features of tissue-resident memory T (T_RM_) cells that expressed high levels of effector proteins. In contrast, TG of "non-protected" SYMP HLA Tg rabbits with increased virus reactivation from TG and increased severity of recurrent ocular herpes, contain a high number of dysfunctional HSV-specific CD8^+^ T_EM_ cells and tissue-resident memory CD8^+^ T_RM_ cells, with significant up-regulation of *PD-1*, *LAG-3*, *TIGIT* and *TIM-3* genes. As exhaustion markers are found to be expressed on circulating CD8^+^ T cells in humans, it is likely that a reservoir of circulating memory CD8^+^ T cells immigrate into nonlymphoid tissues, including TG, which may trigger the observed exhaustion. Similar studies by Klonowski et al*.*, using parabiosis experiments, have demonstrated that circulating memory CD8^+^ T cells, in a resting host, comprised a pool of cells, which were capable of relocating into nonlymphoid tissues^43^. Due to the ethical and practical limitations in obtaining human TG samples from HSV-1-infected SYMP and ASYMP individuals, it was not possible to study exhaustion of tissue-resident HSV-specific CD8^+^ T cells in humans. Instead, we used our established HLA Tg rabbits' model of ocular herpes to demonstrate increased expression of "functional exhaustion" markers on TG-resident CD8^+^ T cells in SYMP HLA Tg rabbits. The report suggests that antiviral CD8^+^ T_EM_ and CD8^+^ T_RM_ cells contribute to herpes immunosurveillance in latently infected TG and their number and function are key targets of a boost by a therapeutic vaccine combined with immune checkpoints blockade (Fig. 7). Beside the central antiviral CXCR3^+^CD8^+^ T_RM_ cells observed in the TG of asymptomatic individuals and rabbits, it is likely that peripheral corneal T cell immunity contribute to protection in asymptomatic individuals and rabbits.

Although our human transcriptome profiling reveals unique molecular characteristics of blood-derived "protective" antiviral CD8^+^ T cells associated with asymptomatic recurrent ocular herpes, it is important to note that the molecular characteristics of blood-derived CD8^+^ T cells may not reflect those of tissue-resident HSV-specific CD8^+^ T cells. In this study, we used our established HLA-A*0201 transgenic rabbit model of recurrent ocular herpes, which develops spontaneous virus reactivation, virus shedding in tears, and symptomatic recurrent ocular herpetic disease, as occurs in humans. Besides, the HLA Tg rabbit model develops human-like CD8^+^ T cell responses to HLA-A*0201 restricted epitopes^3,21,38^, and as such offered the possibility to determine the phenotype, function, and transcriptome of TG-resident HLA-A*0201 restricted HSV-1 epitopes-specific CD8^+^ T cells that would be associated with symptomatic vs. asymptomatic recurrent ocular herpes. This constitutes the first report deciphering the differential gene expression pattern of CD8^+^ T cells in general, and of TG-resident CD8^+^ T cell subsets in particular, in the rabbit model during symptomatic and asymptomatic herpes infection. We observed increased frequencies of functional CD8^+^ T_RM_ cells in TG tissues associated with protection from recurrent ocular herpes in ASYMP HLA Tg rabbits, and identified a conserved tissue signature and activation states for those CD8^+^ T_RM_ cells conserved across many HSV-1 epitopes. Moreover, we demonstrated that high frequencies of functional CD8^+^ T cells in TG explants positively correlated with less virus reactivation ex vivo. Thus, while the antiviral CD8^+^ T cells play a crucial role in reducing HSV-1 reactivations from latently infected TG, the small number of functional CD8^+^ T_RM_ cells present in latently infected TG of SYMPP rabbits (and presumably in TG of SYMP humans) may not be sufficient to prevent virus reactivation^23,44^. In such a scenario, to increase the number of functional TG-resident CD8^+^ T cells, we used the antagonist mAbs approach to block T cell exhaustion pathways. We demonstrate, in both ex vivo in TG explants and in vivo in HSV-1 infected HLA Tg rabbits, that blockade of PD-1 and/or LAG-3 immune checkpoints significantly boosted T-cell immunity associated with a significant reduction in virus reactivation from TG explants and with protection from recurrent ocular herpes infection and disease in HSV-1 latently infected HLA Tg rabbits. To the best of our knowledge, this is a first report using scRNASeq in the rabbit model, providing transcriptional signatures across TG-resident CD45^+^ leukocytes and showed eight transcriptionally distinct major leukocyte subpopulations, CD8^+^ T cells, CD4^+^ T cells, NK cells, B cells, dendritic cells, monocytes, granulocytes, and macrophages. Among these cell populations, we identified increased numbers of CD8^+^ T cells and monocytes as a cellular signature associated with a reduction in recurrent ocular herpes infection and disease in latently infected HLA Tg rabbits.

The profile of biological pathways and genes differentially expressed during herpes infection and disease will aid in the development of therapeutic herpes vaccine. In this study, we found the memory CD8^+^ T_EM_ cells and CD8^+^ T_RM_ cells from TG of "protected" ASYMP HLA Tg rabbits expressed high levels of T cell attracting chemokines/chemokine receptor genes including: *CCL5*, *CCL14*, *CCR7*, and *CXCR3*. The bulk-RNA sequencing also revealed an up-regulation of *CCL2-*, *CCL4-*, *CCL5-*, *CXCL9-*, *CXCL10-*, *CXCL11-*, *CCR7-*, *CXCR3-*, and *VCAM-1-*like genes associated with the chemokine signaling pathway. The HSV-specific CD8^+^ T_EM_ cells and CD8^+^ T_RM_ cells infiltrating the TG of protected HLA Tg rabbits heightened expression of inflammatory chemokines *CCL2*, *CCL4*, *CCL5*, *CXCL9*, and *CXCL10*^45,46^. Recent studies reporting high transcript levels of *CCL4* in quiescent T_RM_ cells associate the phenomenon with the production of such chemokines by T_RM_ cells^47^. These chemokines may subsequently trigger the attraction of neutrophils and monocytes like innate myeloid cells to the site of infection, and further enhance the immune response^48,49^. Similarly, memory CD8^+^ T cells from TG of "protected" ASYMP HLA Tg rabbits expressed high levels of CXCR3, a receptor known to be associated with recruitment of T cells to infected tissues in response to CXCL9- and CXCL10 chemokines^50^. In contrast, a down-regulation of the CAM pathway, which is comprised of major T cell exhaustion molecules, in HSV-specific CD8^+^ T cells was associated with asymptomatic herpes in both humans and HLA Tg rabbits.

We further observed an up-regulation of genes *IL7R*, *IL15R*, *IL17R*, *IL6ST* (*CD130*), and *TNFRSF9* (*CD137*) cytokines and cytokine receptors in the CD8^+^ T_RM_ cells from TG of ASYMP HLA Tg rabbits. In other systems, T_RM_ cells associated to protection upregulate receptors for IL-7 and IL-15^51,52^. Moreover, *IL7R* and *IL15R* contribute to antigen-independent maintenance of T_RM_ cells^51,52^ and the continued presence of IL-15 aids in long-term maintenance of T_RM_ cells in infected tissues^52^. *TNFRSF9* (*CD137*) helps clonal expansion, survival and development of T cells. In both mice and humans, T_RM_ cells across different tissues express higher levels of transcript for the pro-inflammatory cytokines compared to their circulating counterparts^53,54^. Once stimulated by the virus, such as after HSV-1 reactivation from TG, the local CD8^+^ T_RM_ cells release pro-inflammatory cytokines, IFN-*γ*, TNF-α, and IL-2, which amplifies the present several other tissue-resident immune cell, such as the monocytes present with high frequencies in TG of protected HLA Tg rabbits.

We detected an increased frequency of functional HSV-specific CD103^+^CD69^+^CD8^+^ T_RM_ cells with activation pathway-specific genes, such as CD69, CD62L (SELL), and CD44, in the TG of "protected" ASYMP HLA Tg rabbits compared to TG of "non-protected" SYMP HLA Tg rabbits. Our bulk RNA sequencing results also revealed up-regulation of the T cell activation pathway with increased expression of *CD69*, *CD62L* (*SELL*) and *IFN-γ* genes in ASYMP HLA Tg rabbits. This finding is in light of previous reports of increased frequencies of CD8^+^ T_RM_ cells expressing CD69 and/or CD103 in protected tissues of many systems^54–61^. Interestingly, the single cell and bulk RNA-sequencing data showed increased expression of central memory marker *CD62L* (*L-selectin*) in TG-resident memory cells. It appears that the expression of *CD62L* on the surface on the T-cell (at the protein level) is regulated at both the transcriptional and post-transcriptional level. *CD62L* expressed on T cells plays a crucial role during recirculation of central memory T cells (T_CM_ cells), especially into peripheral lymph nodes and are also involved in mediating entry of T_CM_ cells into infected non-lymphoid tissues, such as TG^62^. T cells in non-lymphoid organs such as lung, intestine and liver have been shown to have more *CD62L* expression^63^. Thus, the presence of CD62L^(+)^CD8^+^ T_CM_ cells in TG suggests a migration of HSV-specific T_CM_ cells from periphery into infected TG. The HSV-specific CD69^+^CD8^+^ T_RM_ cells from TG of "protected" ASYMP rabbits with less virus reactivation appeared to be multifunctional, proliferating and producing high levels of CD107 and IFN-*γ*. In agreement with previous reports that rapid control of viral replication is associated to the abundant IFN-*γ* production by cytotoxic CD8^+^ T_RM_ cells^64^. Local CD8^+^ T_RM_-derived IFN-*γ* is responsible for increasing the expression of the homing molecule vascular cell adhesion molecule 1 (VCAM-1) on endothelial cells, and is also known to enhance the recruitment of other memory T cells, such CD8^+^ T_RM_ cells from circulation^45,46^.

We found RNA sequencing of CD8^+^ T cells specific to several HSV-1 epitopes provide a high-resolution map that defined T cell states associated with protective vs. pathogenic recurrent ocular herpes infection^31^. Using RNA profiling in humans and rabbits, we identified a module of co-inhibitory receptors that includes genes for several known co-inhibitory receptors (*PD-1*, *TIM-3*, *LAG-3* and *TIGIT*). Sustained overexpression of co-inhibitory receptors, such as PD-1 and LAG-3, on antiviral CD8^+^ T cells promote T cell dysfunction or exhaustion, leading to impaired ability to clear virus reactivation, as reported in other systems^65–72^. Such T cell dysfunction may contribute to suboptimal herpes immunity and subsequently to a symptomatic virus shedding seen in SYMP patients and SYMP HLA Tg rabbits^31,44^. Thus, even in the presence of a higher frequency of CD8^+^ T_RM_ cells in the TG, as an immune evasion strategy the HSV-1 can still manage to reactivate by inducing exhaustion of these TG-resident CD8^+^ T_RM_ cells^3,6,34^. We phenotypically and functionally validated two co-inhibitory receptors PD-1 and LAG-3 in HSV-1 infected HLA Tg rabbits, where the TG-resident CD8^+^ T cells exhibited transcriptional features of exhausted CD8^+^ T cells. We observed an increased frequency of LAG-3^+^CD8^+^ T cells and PD-1^+^CD8^+^ T cells in "non-protected" SYMP HLA Tg rabbits in comparison to increased frequency of functional LAG-3^-^CD8^+^ T cells and PD-1^-^CD8^+^ T cells in TG of "protected" ASYMP HLA Tg rabbits. It is likely that similar to "non-protected" SYMP HLA Tg rabbits, exhausted CD8^+^ T_RM_ cells in HSV-1 latently infected TG occurs in TG of SYMPP patients. Exhaustion of HSV-specific CD8^+^ T cells may occur during multiple productive replication attempts and/or repetitive virus reactivations, events that might be underestimated with actual molecular detections^73^. Moreover, we found that LAG-3 and PD-1 immune checkpoints blockade in HSV-1 latently infected HLA Tg rabbits restored T cell functions associated with less recurrent ocular herpes infection and disease, confirming our previous studies^18,28,31,35^. The findings in this report prompt to question whether HSV-1 infections can be categorized as a latent or chronic infection.

Our transcriptome profiling data identified the transcription factors *ICOS* and *PRDM1* (*BLIMP-1*) as cooperative regulators of the co-inhibitory (exhaustion) module identified in antiviral CD8^+^ T cells associated with asymptomatic herpes infection. The inducible T cell co-stimulator (*ICOS*) has been reportedly associated with induction of Th1, Th2, Th17 immunity and regulation of effector T cell differentiation and with a role in adaptive immunity^74^. Remarkably, an up-regulation of *ICOS* has been reported in human and mice CD8^+^ T_RM_ cells^54,75^. Similarly, Mackay et al*.* observed that *BLIMP-1* expression by memory T cells was highest eight days after herpes infection and later was maintained at a lower level in memory T cells in the skin^53^. They further suggested that *BLIMP-1* expression is required to form short-lived effector T cells and *BLIMP-1* along with *HOBIT* are necessary for the development of T_RM_ cells but not of circulating memory T cells, suggesting that *BLIMP-1* and *HOBIT* cooperate to promote T_RM_ cell development^53^. Our results provide insights into the transcriptional regulation that influence memory formation and CD8^+^ T cell protective immunity that contribute to asymptomatic herpes. Among the limitations are that the select tissues from SYMP and ASYMP herpes patients profiled in this study may not include the full diversity of T cell transcriptional programs throughout the body, and that quantification of various memory T cell subsets may be subject to dissociation biases between the individual tissue compartments.

In summary, our study demonstrates that HSV-1 infected ASYMP humans and "protected" ASYMP HLA Tg rabbit model have unique transcriptomic, phenotypic and functional characteristics in their antiviral CD8^+^ T cells. Bulk and scRNASeq profiling of HSV-specific memory CD8^+^ T cells from blood and TG, the site of latent infection, demonstrate that high-resolution map of antiviral T cells in symptomatic and asymptomatic herpes serve as a new baseline for defining the coding genes and the immunological pathways that are differentially expressed in CD8^+^ T_RM_ cells during HSV-1 symptomatic vs. asymptomatic infections. Future studies will address the biological mechanisms that regulate "protective" and "pathogenic" pathways in CD8^+^ T_RM_ cells during HSV-1 symptomatic vs. asymptomatic infections. Importantly, this work strengthens the current strategy of developing T-cell based immunotherapeutic approaches to protect from recurrent herpes.

## Methods

### Human study population

All clinical investigations in this study were conducted according to the Declaration of Helsinki. All subjects were enrolled at the University of California, Irvine under approved Institutional Review Board protocols (IRB#2003-3111 and IRB#2009-6963). Written informed consent was received from all participants prior to inclusion in this study.

During the last 17 years (i.e. January 2003–January 2020), we screened 955 individuals for HSV-1 and HSV-2 seropositivity. Since HSV-1 is the main cause of ocular herpes, individuals who were HSV-1 seropositive were enrolled in this study. The HSV-1-seropositive individuals were divided into two groups: (1) HLA-A*02:01-positive ASYMP individuals who, despite being infected, never had any clinically detectable herpes disease; and (2) HLA-A*02:01-positive SYMP individuals with a history of numerous episodes of clinically documented recurrent ocular herpes diseases, such as herpetic lid lesions, herpetic conjunctivitis, dendritic or geographic keratitis, stromal keratitis, and iritis consistent with rHSK, with one or more episodes per year for the past 5 years. Only SYMP patients who were not on Acyclovir or other antiviral or anti-inflammatory drug treatments at the time of blood sample collections were enrolled^16,22,26,76^. SYMP and ASYMP groups were matched for age, gender, serological status, and race. Among the SYMP and ASYMP individuals, 4 HLA-A*02:01-positive patients (ASYMP: *n* = 2, and SYMP: *n* = 2) were enrolled in this study for transcriptome profiling through bulk RNA sequencing. More importantly, as present study was aimed to study the differentially expressed genes in HSV-specific CD8^+^ T cells in ASYMP and SYMP individuals. We have observed none or very few tetramer-positive cells in the seronegative control group, which was not enough to run the RNA sequencing experiment further. Therefore, we did not include seronegative individuals as baseline control in this study.

### HSV specific serotyping

The sera collected from random donors were tested for anti-HSV antibodies. ELISA was performed on sterile 96-well flat-bottom microplates coated with the HSV-1 antigen in coating buffer overnight at 4 °C. The reaction was terminated by adding 1 M H_2_SO_4_. The absorbance was measured at 450 nm.

### Peripheral blood mononuclear cells

Individuals (negative for HIV, HBV, and with or without any HSV infection history) were recruited at the UC Irvine Institute for Clinical and Translational Science (ICTS). One hundred mL of blood was drawn into yellow-top Vacutainer Tubes (BECTON DICKINSON, CA, USA). The serum was isolated and stored at − 80 °C for the detection of anti-HSV-1 and anti-HSV-2 antibodies, as we previously described^77^. PBMCs were isolated by gradient centrifugation using lymphocyte separation medium (CORNING, Tewksbury, MA, USA), subsequently washed in PBS and resuspended in complete culture medium consisting of RPMI1640, 10% FBS (Bio-Products, Woodland, CA, USA) supplemented with 1 × penicillin/streptomycin/l-glutamine, 1 × sodium pyruvate, 1 × non-essential amino acids, and 50 μm of 2-mercaptoethanol (INVITROGEN, Carlsbad, CA, USA).

### Peptide synthesis

HLA-A*0201 binding peptides (9-mer long) corresponding to immunodominant human CD8^+^ T cell epitopes from two HSV-1 proteins gB_561–569_ (RMLGDVMAV), and VP11/12_702–710_ (ALSALLTKL) were synthesized using solid-phase peptide synthesis and standard 9-fluorenylmethoxycarbonyl technology (PE APPLIED BIOSYSTEMS, Foster City, CA, USA). The purity of peptides was over 90%, as determined by reversed-phase high-performance liquid chromatography (Vydac C18) and mass spectroscopy (VOYAGER MALDI-TOF System). Stock solutions were made at 1 mg/mL in 10% DMSO in PBS.

### HLA transgenic rabbits

An HLA transgenic rabbit colony was bred at UC Irvine^19,49^ and the New Zealand White (NZW) rabbits, purchased from Western Oregon Rabbit Co., were used for all the experiments. All rabbits were housed and treated in accordance with ARVO (Association for Research in Vision and Ophthalmology), AAALAC (Association for Assessment and Accreditation of Laboratory Animal Care), and NIH (National Institutes of Health) guidelines. A colony of human leukocyte antigens (HLA) transgenic (Tg) rabbits were maintained at UC Irvine. The HLA Tg rabbits retain their endogenous rabbit major histocompatibility complex (MHC) locus and express human HLA-A*02:01 under the control of its normal promoter^44,78^. Prior to this study, the expression of HLA-A*02:01 molecules on the PBMC of each HLA-Tg rabbit was confirmed by fluorescence-activated cell sorting (FACS) as described previously^10^.

### Herpes simplex virus production and ocular herpes infection

The HSV-1 (lab strain McKrae) was used in this study. The virus was triple plaque purified and prepared as previously described^49,55^. Two groups (Group-1: *n* = 10; Group-2: *n* = 10) of HLA Tg rabbits (8–10 weeks) received an ocular HSV-1 challenge (2 × 10^5^ pfu, lab McKrae strain) without scarification. Following ocular infection, rabbits from both groups were monitored for ocular herpes, virus infection, and disease^44,78^. The Group-1 HSV-1 infected rabbits were observed with no recurrent corneal herpes and categorized as asymptomatic (ASYMP). In contrast, the Group-2 HSV-1 infected rabbits were found with severe corneal herpetic disease and classified as symptomatic (SYMP). On the severity of disease scoring scale ranging between 0 and 4; the ASYMP rabbits scored 0–1, whereas the SYMP rabbits scored 2–4.

### Immune checkpoints blockade

Cross-reactive anti-*PD-1* mAb (RMPI-14) and anti-*LAG-3* mAb (C9B7W) were purchased from BIOXCELL (West Lebanon, NH). NZW and HLA Tg rabbits were ocularly infected with 2 × 10^5^ pfu of lab strain McKrae and received an intravenous injection of 200 μg of anti-*PD-1* mAb and/or anti-*LAG-3* mAb on scheduled days, as illustrated in Fig. 2a.

### Detection of rabbit ocular infectious virus

Tears were collected from both eyes using a Dacron swab (type 1) (SPECTRUM LABORATORIES, CA, USA) following the commencement of blockade post-challenge. Individual swabs were transferred to a 2 mL sterile cryogenic vial containing 1 mL culture medium and stored at − 80 °C until use. The HSV-1 titers in tear samples were determined by standard real-time PCR based on previously described reaction conditions^79^.

### Ex-vivo culture to detect virus reactivation (HSV-1 DNA copies) in Trigeminal Ganglia

Trigeminal ganglion (TG) were harvested HSV-1 infected HLA Transgenic rabbits. The TG were excised, pooled, and finely chopped and placed into the culture with RPMI 1640 medium + 2% FBS. Cultures in duplicates were then incubated at 37 °C for 10 days with 100 µg/mL of blocking mAbs, targeting mPD-1 (clone J43; BIOXCELL), and mLAG-3 (clone C9B7W; BIOXCELL). On days 4, 6, 8 post infection, the mAbs were replenished and 600 µL of culture supernatant fluid was removed from each culture, stored at − 80 °C for the virus titration, and replaced this with an equal volume of fresh medium. The supernatant from TG was assayed for infectious HSV-1 by real-time PCR^79^.

### Detection of rabbit corneal herpetic disease

Rabbits were examined for ocular disease and survival for 30 days following blockade mAbs treatment after the infection. The ocular disease was determined by a masked investigator, and pictures were taken before the infection and following the blockade post-infection. For disease scoring we used a standard 0–4 scale: 0, no disease; 1, 25%; 2, 50%; 3, 75%; and 4, 100% staining, was used.

### Bulk RNA-sequencing

HSV-specific CD8^+^ T cells were FACS sorted from PBMC of ASYMP and SYMP human subjects using tetramers specific to immunodominant epitopes selected from both glycoprotein and tegument proteins: (1) glycoprotein gB (gB_561–569_); and (2) tegument protein VP11/12 (VP11/12_702–710_). Post-sorting acquisition was performed for the purpose of post-sort purity check (Supplementary Fig. S2). Similarly, TG of blockade-treated and mock-treated HLA Tg rabbits were treated using tetramers specific to immunodominant epitopes selected from both glycoproteins and tegument proteins: (1) the glycoprotein gB (gB_183–191_); and (2) the tegument protein VP11/12 (VP11/12_702–710_). RNA was isolated from sorted cells using Direct-zol RNS MiniPrep (ZYMO RESEARCH, Irvine, CA, USA) according to the manufacturer's instructions. Agilent 2100 Bioanalyzer was used to calculate the RNA concentration, and integrity. We constructed sequencing libraries using TruSeq Stranded Total RNA Sample Preparation Kit (ILLUMINA, San Diego, CA, USA). Briefly, rRNA-depleted RNA was fragmented, converted to ds cDNA, and ligated to adapters, amplified by PCR, and selected by size exclusion. Following quality control for size, quality, and concentrations, libraries were multiplexed and sequenced to single-end 100 bp sequencing using the Illumina HiSeq 4000 platform.

### Droplet-based scRNASeq

For droplet-based scRNASeq, libraries were prepared from CD45^+^ T cells sorted from the TG of ASYMP (HLA Tg rabbits treated with α-*PD-1* and α-*LAG-3* mAbs) and SYMP (HLA Tg rabbits not subjected to *PD-1* and *LAG-3* blockade treatment) HLA Tg rabbits using the Chromium Single Cell 3′ Reagent Kits v.3 according to the manufacturer's protocol (10 × GENOMICS). The generated scRNASeq libraries were sequenced using Hiseq 4000 (150 cycles).

### Analysis of droplet-based scRNASeq

Gene counts were obtained by aligning reads to the rabbit genome assembly OryCun2.0 (GCA_000003625.1) using Cell Ranger software (v.3.0.2) (10 × Genomics). A gene-count matrix was generated after quantification of mRNA using the Cell Ranger count pipeline. The filtered gene count matrix files were used for downstream analysis using Seurat 3.0. Then, a cut-off value of 200 unique molecular identifiers (UMIs) was used to select single cells for further analysis. This resulted in an initial dataset that was subsequently examined to exclude low-quality libraries^80^.

### Quality control for cell inclusion

The initial dataset contained 906 cells for ASYMP group and 886 cells for SYMP group of HLA Tg rabbits. *t*-distributed stochastic neighbor embedding (*t*-SNE) was used to project the entire dataset onto the two-dimensional space based on the top 10 principal components. For each cell, we evaluated; (1) the number of genes for which at least one read was mapped (which is indicative of library complexity), (2) the total number of counts, (3) the percentage of counts mapping to the top 50 genes, and (4) the percentage of reads mapped to mitochondrial genes. Cells showing a relatively lower proportion of endogenous RNAs might suggested that either the cells were stressed or dead and resulted in RNA degradation. Outlier cells in these quality metrics were found to cluster together in the *t*-SNE two-dimensional space. Further, *k*-means clustering algorithm was applied and cells with an abnormally high ratio of counts mapped to mitochondrial genes were removed and gene counts associated with protein-coding genes were included for downstream analysis. At the end of quality control, 902 cells among ASYMP and 745 cells among SYMP groups remained.

### Cell clustering and marker identification

Data normalization and clustering was performed on quality control passed ASYMP (n = 902) and SYMP (n = 745) cells with the Seurat 3.0. Counts for all were scaled by the total library size multiplied by 10,000 and transformed to log space. A total of 3,078 and 2,964 highly variable genes for ASYMP and SYMP groups were identified based on mean and dispersion, and the values were rescaled. Principal component analysis (PCA) was performed on the variable genes, and *t*-SNE was run on the top 10 principal components (PCs) and the Louvain graph-clustering algorithm was applied to identify cell clusters. For each cluster, we assigned a cell type label using statistical enrichment for sets of marker genes and manual evaluation of gene expression for small sets of known marker genes (CD8^+^ T) cell (CD8A), CD4^+^ T cell (CD4), NK cell (NKG7), B cell (CD19), Macrophage (CD68), Monocyte (CD14), Granulocyte (FUT4), and, Dendritic cell (CD1c). Significantly overexpressed genes were defined based on the Wilcoxon rank-sum test with FDR-corrected *P* value ≤ 0.01.

### Differential gene expression analysis

The differentially expressed genes (DEGs) were analyzed by using integrated Differential Expression and Pathway analysis (iDEP) tool^81^. iDEP is a comprehensive analysis toll comprising of 63 R/Bioconductor packages, two web services, and well-annotated pathway databases for multiple animal species including that of humans and rabbits. The gene count data obtained after applying quality control parameters were subject to DEG analysis. The genes were converted to Ensemble gene IDs and filtered based on gene count. Subsequently, the pre-processed data was used for *k*-means and hierarchical clustering. The pairwise comparison of human and rabbit (ASYMP vs. SYMP) was performed using the DESeq2 package with a threshold of false discovery rate (FDR) < 0.5. and fold-change > 1.5. Although DESeq, SAMseq, EBSeq methods can be used to identify differentially expressed genes (DEG) in RNASeq data, in this study, we preferred DESeq over the other approaches because it allows obtaining better results with small samples (e.g., two samples per condition as used in this study), as reported previously^82,83^. Unlike the DESeq approach used in this study, the results obtained by other approaches, such as SAMseq, can be influenced by the small sample size and may produce many false positives^84^. Similarly, in scRNA Seq, the statistical power comes from the analysis of multiple cells rather than biological samples, which justifies the use of two samples per group, as previously reported^85^. Moreover, a hierarchical clustering tree and network of enriched GO/KEGG terms were constructed to visualize the potential relationship. Gene set enrichment analysis (GSEA) method was performed to investigate the related signal pathways activated among ASYMP and SYMP groups. Parametric gene set enrichment analysis (PSGEA) method was applied based on data curated in Gene Ontology and KEGG. Pathway significance cutoff with a false discovery date (FDR) ≥ 0.2 was applied.

### Flow cytometry assays

Trigeminal ganglia were analyzed by flow cytometry, as we previously reported^86^. The following antibodies were used: mouse anti-rabbit CD8 (clone MCA1576F, SEROTEC), mouse anti-human CD103 (clone H4A3) FITC, CD69 (clone H4B4) APC/Cy7 (BIOLEGEND), and rat anti-IFN-*γ* (clone XMG1.2) (BD Biosciences). For surface staining, mAbs against various cell markers were added to a total of 1 × 10^6^ cells in phosphate-buffered saline containing 1% FBS and 0.1% sodium azide (FACS buffer) and left for 45 min at 4 °C. For intracellular staining, mAbs were added to the cells and incubated for 45 min on ice and in the dark. A total of 50,000 events were acquired by LSRII (BECTON DICKINSON, Mountain View, CA, USA), followed by analysis using FlowJo software (TREESTAR, Ashland, OR, USA).

### Immunohistochemistry

Rabbit TG was cut into 8 µm thick sections using a cryostat. Sections were washed with 1× PBS, permeabilized using 0.05% Triton X 100 in 1× PBS for 15 min, and blocked using 10% FBS in 1× PBS for 1 h. Sections were stained using anti-rabbit CD8^+^, CXCR3, CXCL9, CXCL10, CXCL11 antibodies (1:200) overnight at 4 °C BD Pharmingen, Inc., San Diego, CA, USA). After secondary fluorescent staining, sections were washed with 1× PBS and mounted after DAPI staining (1:10,000 dilution). Immunofluorescence infiltration of CD8^+^ T cells was examined using a Keyence BZ-X700 fluorescent microscope at 40 × magnification and imaged using z-stack.

### Statistical analyses

Data for each differentially expressed gene among blockade-treated, and mock-treated groups of HLA Tg rabbits were compared by analysis of variance (ANOVA) and Student's *t* test using GraphPad Prism version 5 (La Jolla, CA, USA). ANOVA and multiple comparison procedures were followed to calculate the statistical differences between the study groups. Data are expressed as the mean ± SD. Results were considered statistically significant at *P* ≤ 0.05.

## Supplementary information

Supplementary Figure S1.

Supplementary Figure S2.

Supplementary Tables.

Supplementary Legends.

## References

[1] Looker KJ (2015). Global and regional estimates of prevalent and incident herpes simplex virus Type 1 infections in 2012. PLoS One.

[2] Mott KR (2009). Level of herpes simplex virus type 1 latency correlates with severity of corneal scarring and exhaustion of CD8+ T cells in trigeminal ganglia of latently infected mice. J. Virol..

[3] Chentoufi AA (2010). A novel HLA (HLA-A*0201) transgenic rabbit model for preclinical evaluation of human CD8+ T cell epitope-based vaccines against ocular herpes. J. Immunol..

[4] Chentoufi AA (2008). HLA-A*0201-restricted CD8+ cytotoxic T lymphocyte epitopes identified from herpes simplex virus glycoprotein D. J. Immunol..

[5] Allen SJ (2011). The role of LAT in increased CD8+ T cell exhaustion in trigeminal ganglia of mice latently infected with herpes simplex virus 1. J. Virol..

[6] Knickelbein JE (2008). Noncytotoxic lytic granule-mediated CD8+ T cell inhibition of HSV-1 reactivation from neuronal latency. Science.

[7] Koujah L, Suryawanshi RK, Shukla D (2019). Pathological processes activated by herpes simplex virus-1 (HSV-1) infection in the cornea. Cell. Mol. Life Sci..

[8] Agelidis AM, Shukla D (2015). Cell entry mechanisms of HSV: What we have learned in recent years. Future Virol..

[9] Harrison KS, Zhu L, Thunuguntla P, Jones C (2019). Antagonizing the glucocorticoid receptor impairs explant-induced reactivation in mice latently infected with herpes simplex virus 1. J. Virol..

[10] Watson ZL (2018). In vivo knockdown of the herpes simplex virus 1 latency-associated transcript reduces reactivation from latency. J. Virol..

[11] BenMohamed L (2016). Prior corneal scarification and injection of immune serum are not required before ocular HSV-1 infection for UV-B-induced virus reactivation and recurrent herpetic corneal disease in latently infected mice. Curr. Eye Res..

[12] BenMohamed L (2015). Decreased reactivation of a herpes simplex virus type 1 (HSV-1) latency-associated transcript (LAT) mutant using the in vivo mouse UV-B model of induced reactivation. J. Neurovirol..

[13] Roizman B, Whitley RJ (2013). An inquiry into the molecular basis of HSV latency and reactivation. Annu. Rev. Microbiol..

[14] Chentoufi AA, Kritzer E, Yu DM, Nesburn AB, Benmohamed L (2012). Towards a rational design of an asymptomatic clinical herpes vaccine: The old, the new, and the unknown. Clin. Dev. Immunol..

[15] Dasgupta G (2012). Immunodominant "asymptomatic" herpes simplex virus 1 and 2 protein antigens identified by probing whole-ORFome microarrays with serum antibodies from seropositive asymptomatic versus symptomatic individuals. J. Virol..

[16] Dervillez X (2013). Asymptomatic HLA-A*02:01-restricted epitopes from herpes simplex virus glycoprotein B preferentially recall polyfunctional CD8+ T cells from seropositive asymptomatic individuals and protect HLA transgenic mice against ocular herpes. J. Immunol..

[17] Khan AA (2014). Asymptomatic memory CD8+ T cells: From development and regulation to consideration for human vaccines and immunotherapeutics. Hum. Vaccin. Immunother..

[18] Khan AA (2015). Therapeutic immunization with a mixture of herpes simplex virus 1 glycoprotein D-derived "asymptomatic" human CD8+ T-cell epitopes decreases spontaneous ocular shedding in latently infected HLA transgenic rabbits: Association with low frequency of local PD-1+ TIM-3+ CD8+ exhausted T cells. J. Virol..

[19] Khan AA (2015). Phenotypic and functional characterization of herpes simplex virus glycoprotein B epitope-specific effector and memory CD8+ T cells from symptomatic and asymptomatic individuals with ocular herpes. J. Virol..

[20] Srivastava R (2015). A herpes simplex virus type 1 human asymptomatic CD8+ T-cell epitopes-based vaccine protects against ocular herpes in a "humanized" HLA transgenic rabbit model. Invest. Ophthalmol. Vis. Sci..

[21] Srivastava R (2015). Herpes simplex virus type 1 human asymptomatic CD8 T cell epitopes protect against ocular herpes in "humanized" HLA transgenic rabbit model. IOVS.

[22] Srivastava R (2015). HLA-A02:01-restricted epitopes identified from the herpes simplex virus tegument protein VP11/12 preferentially recall polyfunctional effector memory CD8+ T cells from seropositive asymptomatic individuals and protect humanized HLA-A*02:01 transgenic mice against ocular herpes. J. Immunol..

[23] Srivastava R (2016). Human asymptomatic epitopes identified from the herpes simplex virus tegument protein VP13/14 (UL47) preferentially recall polyfunctional effector memory CD44highCD62LlowCD8+ TEM cells and protect "humanized" HLA-A*02:01 transgenic mice against ocular herpes. J. Virol..

[24] Srivastava R (2017). Human asymptomatic epitopes identified from the herpes simplex virus tegument protein VP13/14 (UL47) preferentially recall polyfunctional effector memory CD44high CD62Llow CD8+ TEM cells and protect humanized HLA-A*02:01 transgenic mice against ocular herpesvirus infection. J. Virol..

[25] Vahed H (2018). Unique type i interferon, expansion/survival cytokines and JAK/STAT gene signatures of multi-functional HSV-specific effector memory CD8(+) TEM cells are associated with asymptomatic ocular herpes in humans. J. Virol..

[26] Vahed H (2019). Unique Type I interferon, expansion/survival cytokines, and JAK/STAT gene signatures of multifunctional herpes simplex virus-specific effector memory CD8(+) TEM cells are associated with asymptomatic herpes in humans. J. Virol..

[27] Chentoufi AA (2012). The herpes simplex virus type 1 latency-associated transcript inhibits phenotypic and functional maturation of dendritic cells. Viral. Immunol..

[28] Chentoufi AA (2011). The herpes simplex virus 1 latency-associated transcript promotes functional exhaustion of virus-specific CD8+ T cells in latently infected trigeminal ganglia: A novel immune evasion mechanism. J. Virol..

[29] Frank GM (2010). Early CD4(+) T cell help prevents partial CD8(+) T cell exhaustion and promotes maintenance of herpes simplex virus 1 latency. J. Immunol..

[30] Allen SJ, Mott KR, Zandian M, Ghiasi H (2010). Immunization with different viral antigens alters the pattern of T cell exhaustion and latency in herpes simplex virus type 1-infected mice. J. Virol..

[31] Roy S (2018). Blockade of LAG-3 immune checkpoint combined with therapeutic vaccination restore the function of tissue-resident anti-viral CD8(+) T cells and protect against recurrent ocular herpes simplex infection and disease. Front. Immunol..

[32] Rutkowski AJ (2015). Widespread disruption of host transcription termination in HSV-1 infection. Nat. Commun..

[33] Cheung P, Panning B, Smiley JR (1997). Herpes simplex virus immediate-early proteins ICP0 and ICP4 activate the endogenous human alpha-globin gene in nonerythroid cells. J. Virol..

[34] Hoshino Y, Pesnicak L, Cohen JI, Straus SE (2007). Rates of reactivation of latent herpes simplex virus from mouse trigeminal ganglia ex vivo correlate directly with viral load and inversely with number of infiltrating CD8+ T cells. J. Virol..

[35] Roy S (2019). Blockade of PD-1 and LAG-3 immune checkpoints combined with vaccination restore the function of anti-viral tissue-resident CD8(+) TRM cells and reduce ocular herpes simplex infection and disease in HLA transgenic rabbits. J. Virol..

[36] Pesola JM, Zhu J, Knipe DM, Coen DM (2005). Herpes simplex virus 1 immediate-early and early gene expression during reactivation from latency under conditions that prevent infectious virus production. J. Virol..

[37] Liu T, Khanna KM, Chen X, Fink DJ, Hendricks RL (2000). CD8(+) T cells can block herpes simplex virus type 1 (HSV-1) reactivation from latency in sensory neurons. J. Exp. Med..

[38] Khan AA (2015). Therapeutic immunization with a mixture of herpes simplex virus type 1 glycoprotein D derived, "asymptomatic" human CD8+ T-cell epitopes decreases spontaneous ocular shedding in latently infected HLA transgenic rabbits: Association with low frequency of local PD-1+TIM-3+CD8+ exhausted T cells. J. Virol..

[39] Yang S, Liu F, Wang QJ, Rosenberg SA, Morgan RA (2011). The shedding of CD62L (L-selectin) regulates the acquisition of lytic activity in human tumor reactive T lymphocytes. PLoS One.

[40] Chen A, Engel P, Tedder TF (1995). Structural requirements regulate endoproteolytic release of the L-selectin (CD62L) adhesion receptor from the cell surface of leukocytes. J. Exp. Med..

[41] Kahn J, Ingraham RH, Shirley F, Migaki GI, Kishimoto TK (1994). Membrane proximal cleavage of L-selectin: Identification of the cleavage site and a 6-kD transmembrane peptide fragment of L-selectin. J. Cell Biol..

[42] Peschon JJ (1998). An essential role for ectodomain shedding in mammalian development. Science.

[43] Klonowski KD (2004). Dynamics of blood-borne CD8 memory T cell migration in vivo. Immunity.

[44] Srivastava R (2016). The herpes simplex virus latency-associated transcript gene is associated with a broader repertoire of virus-specific exhausted CD8+ T cells retained within the trigeminal ganglia of latently infected HLA transgenic rabbits. J. Virol..

[45] Iijima N, Iwasaki A (2014). T cell memory. A local macrophage chemokine network sustains protective tissue-resident memory CD4 T cells. Science.

[46] Schenkel JM, Fraser KA, Vezys V, Masopust D (2013). Sensing and alarm function of resident memory CD8(+) T cells. Nat. Immunol..

[47] Wakim LM (2012). The molecular signature of tissue resident memory CD8 T cells isolated from the brain. J. Immunol..

[48] Reichel CA (2012). C–C motif chemokine CCL3 and canonical neutrophil attractants promote neutrophil extravasation through common and distinct mechanisms. Blood.

[49] Shi C, Pamer EG (2011). Monocyte recruitment during infection and inflammation. Nat. Rev. Immunol..

[50] Mackay LK (2013). The developmental pathway for CD103(+)CD8+ tissue-resident memory T cells of skin. Nat. Immunol..

[51] Thome JJ (2014). Spatial map of human T cell compartmentalization and maintenance over decades of life. Cell.

[52] Mackay LK (2015). T-box transcription factors combine with the cytokines TGF-beta and IL-15 to control tissue-resident memory T cell fate. Immunity.

[53] Mackay LK (2016). Hobit and Blimp1 instruct a universal transcriptional program of tissue residency in lymphocytes. Science.

[54] Kumar BV (2017). Human tissue-resident memory T cells are defined by core transcriptional and functional signatures in lymphoid and mucosal sites. Cell Rep..

[55] Hombrink P (2016). Programs for the persistence, vigilance and control of human CD8(+) lung-resident memory T cells. Nat. Immunol..

[56] Pallett LJ (2017). IL-2(high) tissue-resident T cells in the human liver: Sentinels for hepatotropic infection. J. Exp. Med..

[57] Purwar R (2011). Resident memory T cells (T(RM)) are abundant in human lung: Diversity, function, and antigen specificity. PLoS One.

[58] Thome JJ, Farber DL (2015). Emerging concepts in tissue-resident T cells: Lessons from humans. Trends Immunol..

[59] Watanabe R (2015). Human skin is protected by four functionally and phenotypically discrete populations of resident and recirculating memory T cells. Sci. Transl. Med..

[60] Wong MT (2016). A High-dimensional atlas of human T cell diversity reveals tissue-specific trafficking and cytokine signatures. Immunity.

[61] Woon HG (2016). Compartmentalization of total and virus-specific tissue-resident memory CD8+ T cells in human lymphoid organs. PLoS Pathog..

[62] Rosen SD (2004). Ligands for L-selectin: Homing, inflammation, and beyond. Annu. Rev. Immunol..

[63] Klinger A (2009). Cyclical expression of L-selectin (CD62L) by recirculating T cells. Int. Immunol..

[64] Jiang X (2012). Skin infection generates non-migratory memory CD8+ T(RM) cells providing global skin immunity. Nature.

[65] Barber DL (2006). Restoring function in exhausted CD8 T cells during chronic viral infection. Nature.

[66] Wherry EJ (2007). Molecular signature of CD8+ T cell exhaustion during chronic viral infection. Immunity.

[67] Haining WN (2008). Identification of an evolutionarily conserved transcriptional signature of CD8 memory differentiation that is shared by T and B cells. J. Immunol..

[68] Aubert RD (2011). Antigen-specific CD4 T-cell help rescues exhausted CD8 T cells during chronic viral infection. Proc. Natl. Acad. Sci. USA.

[69] Duraiswamy J (2011). Phenotype, function, and gene expression profiles of programmed death-1(hi) CD8 T cells in healthy human adults. J. Immunol..

[70] Youngblood B (2011). Chronic virus infection enforces demethylation of the locus that encodes PD-1 in antigen-specific CD8(+) T cells. Immunity.

[71] Im SJ (2016). Defining CD8+ T cells that provide the proliferative burst after PD-1 therapy. Nature.

[72] Jadhav RR (2019). Epigenetic signature of PD-1+ TCF1+ CD8 T cells that act as resource cells during chronic viral infection and respond to PD-1 blockade. Proc. Natl. Acad. Sci. USA.

[73] Mueller SN, Ahmed R (2009). High antigen levels are the cause of T cell exhaustion during chronic viral infection. Proc. Natl. Acad. Sci. USA.

[74] Wikenheiser DJ, Stumhofer JS (2016). ICOS co-stimulation: Friend or foe?. Front. Immunol..

[75] Mackay LK, Kallies A (2017). Transcriptional regulation of tissue-resident lymphocytes. Trends Immunol..

[76] Samandary S (2014). Associations of HLA-A, HLA-B and HLA-C alleles frequency with prevalence of herpes simplex virus infections and diseases across global populations: Implication for the development of an universal CD8+ T-cell epitope-based vaccine. Hum. Immunol..

[77] Chentoufi AA (2008). Asymptomatic human CD4+ cytotoxic T-cell epitopes identified from herpes simplex virus glycoprotein B. J. Virol..

[78] Khan AA (2018). Human asymptomatic epitope peptide/CXCL10-based prime/pull vaccine induces herpes simplex virus-specific gamma interferon-positive CD107(+) CD8(+) T cells that infiltrate the corneas and trigeminal ganglia of humanized HLA transgenic rabbits and protect against ocular herpes challenge. J. Virol..

[79] Kessler HH (2000). Detection of Herpes simplex virus DNA by real-time PCR. J. Clin. Microbiol..

[80] Salomon R (2019). Droplet-based single cell RNAseq tools: A practical guide. Lab Chip.

[81] Ge SX, Son EW, Yao R (2018). iDEP: An integrated web application for differential expression and pathway analysis of RNA-Seq data. BMC Bioinform..

[82] Soneson C, Delorenzi M (2013). A comparison of methods for differential expression analysis of RNA-seq data. BMC Bioinform..

[83] Costa-Silva J, Domingues D, Lopes FM (2017). RNA-Seq differential expression analysis: An extended review and a software tool. PLoS One.

[84] Seyednasrollah F, Laiho A, Elo LL (2015). Comparison of software packages for detecting differential expression in RNA-seq studies. Brief Bioinform..

[85] Davis A, Gao R, Navin NE (2019). SCOPIT: Sample size calculations for single-cell sequencing experiments. BMC Bioinform..

[86] Roy S (2019). Blockade of PD-1 and LAG-3 immune checkpoints combined with vaccination restores the function of antiviral tissue-resident CD8(+) TRM cells and reduces ocular herpes simplex infection and disease in HLA transgenic rabbits. J. Virol..

