# Supplementary figures and images for: Unique molecular signatures of antiviral memory CD8+ T cells associated with asymptomatic recurrent ocular herpes
Source: Sci Rep. 2020 Aug 14;10:13843. doi: 10.1038/s41598-020-70673-z (PMC7427992; doi:10.1038/s41598-020-70673-z)

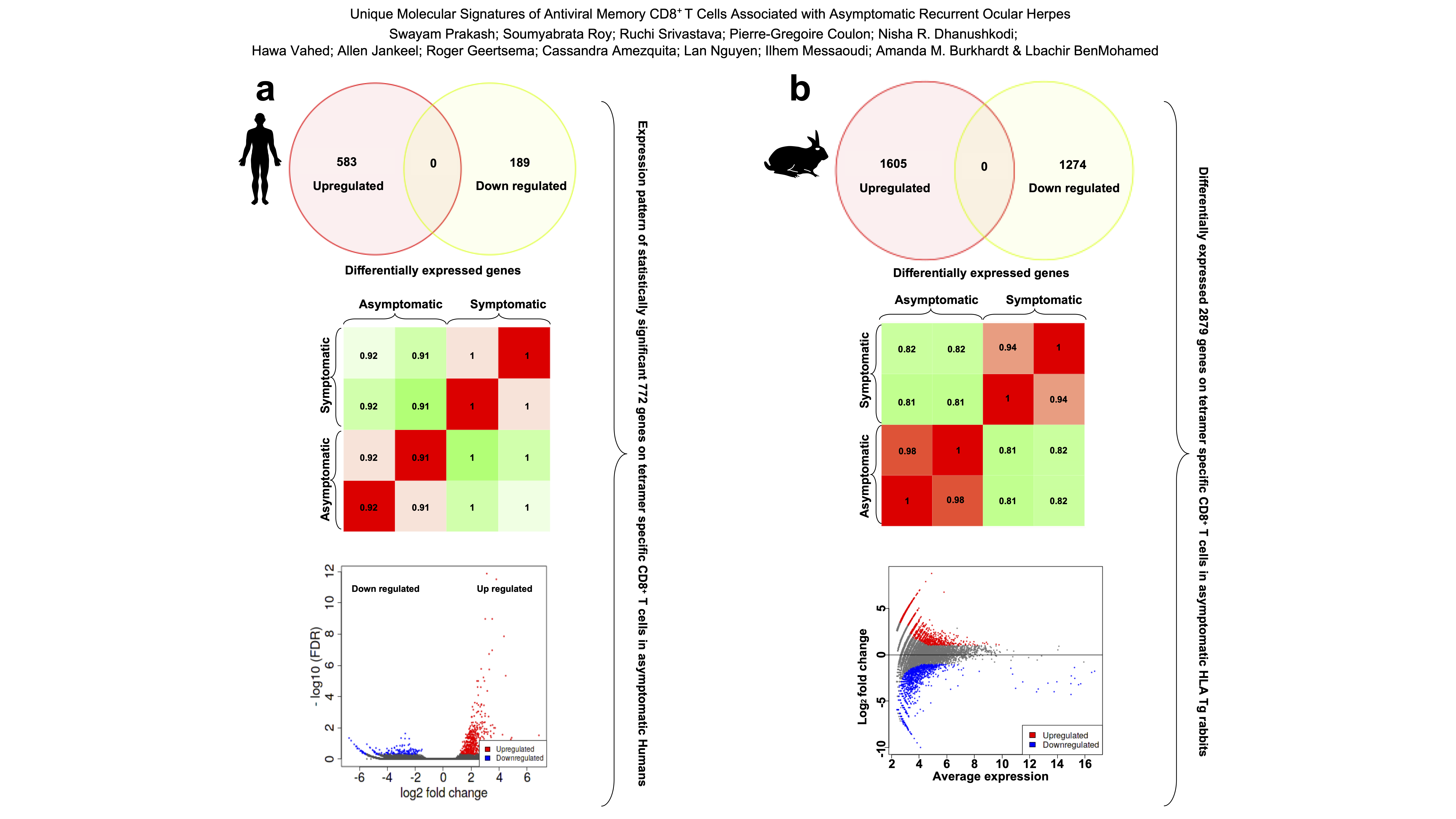

Supplement: Supplementary file 1 — Supplementary Figure S1. [file 41598_2020_70673_MOESM1_ESM.tiff]

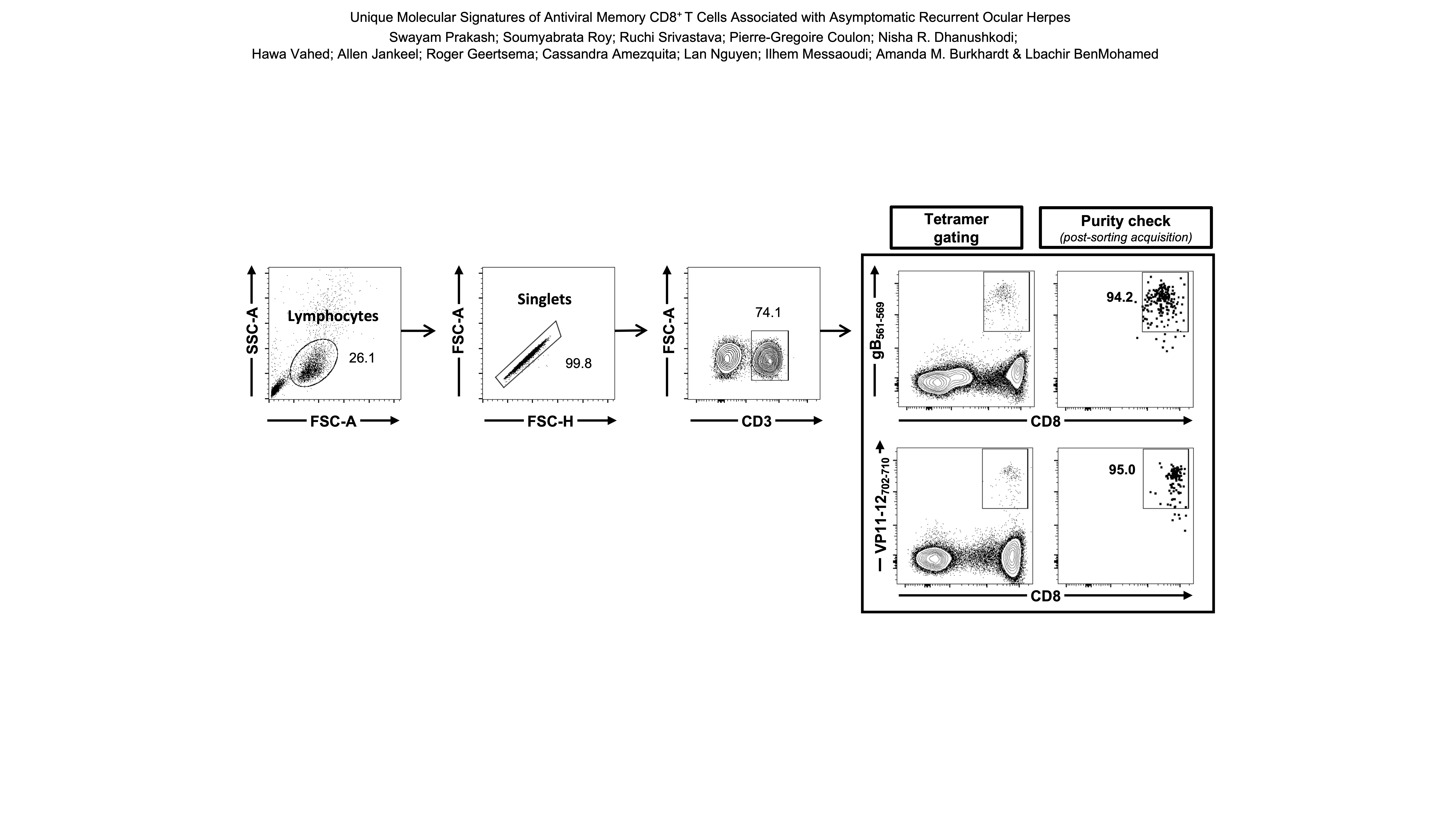

Supplement: Supplementary file 2 — Supplementary Figure S2. [file 41598_2020_70673_MOESM2_ESM.tiff]
