# Supplementary material for: Unique molecular signatures of antiviral memory CD8+ T cells associated with asymptomatic recurrent ocular herpes
Source: Sci Rep. 2020 Aug 14;10:13843. doi: 10.1038/s41598-020-70673-z (PMC7427992; doi:10.1038/s41598-020-70673-z)
Supplement: Supplementary file 3 — Supplementary Tables. [file 41598_2020_70673_MOESM3_ESM.pdf]

Unique Molecular Signatures of Antiviral Memory CD8$^+$ T Cells Associated with Asymptomatic Recurrent Ocular Herpes

Swayam Prakash; Soumyabrata Roy; Ruchi Srivastava; Pierre-Gregoire Coulon; Nisha R. Dhanushkodi; Hawa Vahed; Allen Jankeel; Roger Geertsema; Cassandra Amezquita; Lan Nguyen; Ilhem Messaoudi; Amanda M. Burkhardt & Lbachir BenMohamed

Supplementary Table S1. Differential expression of T cell activation pathway-specific genes in the CD8$^+$ T_{RM} cells, and tissue-resident CD8$^+$ T cell in asymptomatic HLA-Tg rabbits

	Gene expression in CD8$^+$ T_{RM} cells (ASYMP HLA-Tg rabbits)			Gene expression in CD8$^+$ T cells (ASYMP HLA-Tg rabbits)		
**Gene**	**FC**	***P*-value**	**FDR**	**FC**	***P*-value**	**FDR**
IFNG	2.03	0.009	0.01	2.19	0.01	0.05
TNFSF8	2.39	0.01	0.05	2.51	0.03	0.05
BATF	3.12	0.008	0.002	5.74	0.002	0.03
JAML	4.09	0.0005	0.001	3.08	2.14E-05	0.0004
ICOS	4.03	0.006	0.05	4.54	0.002	0.01
CD107	2.14	7.69E-06	7.04E-05	2.31	0.0003	0.002
IRF4	2.02	2.95E-05	0.0002	2.12	0.01	0.05
CD62L	6.48	2.11E-05	0.004	6.90	0.008	0.05
UCHL3	2.07	4.54E-05	0.0009	2.30	0.006	0.02
CD69	2.72	0.002	0.03	7.51	0.003	0.03
CCL5	3.29	4.98E-30	4.13E-26	2.08	0.005	0.01
LAT	4.26	2.00E-03	2.00E-02	2.05	0.004	0.03
CD44	2.79	1.43E-08	1.52E-06	3.25	3.28E-05	1.96E-04
BLIMP1	3.10	0.007	0.01	4.91	0.0009	0.009
RAC2	-1.05	0.008	0.01	-0.85	0.02	0.05
NLRC3	-1.03	7.56E-05	0.0009	-0.76	0.002	0.01
MSN	-2.18	0.0006	0.004	-0.52	0.0002	0.003
TIGIT	-1.43	0.01	0.03	-2.70	0.01	0.05
DDOST	-4.05	8.84E-06	0.00002	-0.95	0.004	0.01
RAB27A	-2.02	0.0004	0.009	-2.67	0.0005	0.004
PSMB10	-1.09	0.003	0.01	-7.13	0.0002	0.002
LAG3	-4.15	0.006	0.04	-2.10	0.005	0.04
TIM3	-3.60	0.003	0.01	-6.49	0.001	0.01

FC: Fold change gene expression; Statistical significance when $P \leq 0.05$; FDR: False discovery rate.

The differential gene expression data shown for CD8$^+$ T_{RM} cells are based on the CD8$^+$ T_{RM} specific normalized gene count data obtained from single-cell RNA sequencing. The CD8$^+$ T cell specific differential gene expression for HLA-Tg rabbits were based on the normalized gene count data obtained from Bulk-RNA sequencing. The ASYMP groups were compared with the gene count data for SYMP groups for the differential gene expression.

Supplementary Table S2. Expression pattern of differentially expressed genes associated with cell adhesion molecules pathway among asymptomatic HLA-Tg rabbits (CD8+ T_{RM} cells, and CD8+ T cell)

	Gene expression in CD8+ T_{RM} cells (ASYMP HLA-Tg rabbits)			Gene expression in CD8+ T cells (ASYMP HLA-Tg rabbits)		
**Gene**	**FC**	**p-value**	**FDR**	**FC**	**p-value**	**FDR**
TIGIT	-1.43	0.01	0.03	-2.70	0.01	0.05
PD1	-2.02	0.0006	0.005	-5.58	1.25E-08	1.01E-06
CTLA4	-1.78	0.003	0.02	-1.04	0.0003	0.01
CD28	-0.08	0.0003	0.01	-0.40	0.004	0.05
CD274	-1.06	0.0007	0.008	-0.27	0.0009	0.01
VCAN	-2.30	0.0007	0.002	-2.84	3.43E-10	4.19E-08
LAG3	-4.15	0.006	0.04	-2.10	0.005	0.04
CD80	-1.07	0.001	0.02	-1.06	0.0003	0.004
L1CAM	-3.23	0.01	0.05	-4.29	2.83E-11	4.61E-09
ITGA4	-0.20	0.007	0.01	-0.61	0.01	0.05
ITGB1	-0.12	0.008	0.04	-0.58	0.0002	0.003
SELE	-5.56	7.68E-06	6.72E-05	-9.71	1.53E-23	1.15E-20
MPZ	-3.29	7.89E-08	6.78E-06	-6.55	7.16E-13	1.54E-10
NCAM1	-1.02	0.0006	0.002	-1.63	0.009	0.05
SELP	-0.02	6.08E-05	0.0009	-3.70	2.49E-10	3.19E-08
CLDN11	-0.02	8.70E-06	2.70E-05	-1.69	0.002	0.01
NRCAM	-2.02	9.68E-07	2.70E-05	-3.12	0.006	0.03
HLA-DRA	-1.02	0.007	0.03	-1.36	4.38E-08	2.98E-06
NLGN3	-4.37	0.006	0.02	-5.12	5.24E-07	2.00E-05
NECTIN1	-0.02	0.002	0.03	-0.79	0.002	0.01
CD226	-3.02	2.95E-05	0.0002	-1.28	0.02	0.05
ITGB7	-0.92	3.80E-06	6.72E-05	-2.15	3.45E-06	9.00E-05
GLG1	-2.32	0.006687569	0.04	-0.88	0.007	0.03
CD62L	2.48	2.11E-05	0.004	2.90	0.008	0.05

CD22	3.02	9.75242E-07	2.70E-05	6.45	0.002	0.01
BLIMP1	3.10	0.007	0.01	4.91	0.0009	0.009
CD4	2.95	0.004	0.03	2.85	0.01	0.05
ICOS	4.03	0.006	0.04	4.54	0.002	0.01
CLDN4	2.05	0.01	0.05	7.11	0.0002	0.002
ITGAV	2.67	0.009	0.02	1.56	0.0007	0.006
PTPRF	3.67	0.008	0.04	6.85	0.001	0.01
PTPRC	2.10	0.001	0.02	2.43	0.0005	0.006
CD40LG	1.58	0.005	0.01	2.32	0.0005	0.006

FC: Fold change gene expression; Statistical significance when $P \leq 0.05$; FDR: False discovery rate.

The differential gene expression data shown for $CD8^+$ T_{RM} cells are based on the $CD8^+$ T_{RM} specific normalized gene count data obtained from single-cell RNA sequencing. The $CD8^+$ T cell specific differential gene expression for HLA-Tg rabbits were based on the normalized gene count data obtained from Bulk-RNA sequencing. The comparison was made between ASYMP vs SYMP groups.

Supplementary Table S3. Chemokine signaling pathway-specific genes differentially expressed in the CD8$^+$ T$_{RM}$ cells, and CD8$^+$ T cell in asymptomatic HLA-Tg rabbit trigeminal ganglia

	Gene expression in CD8$^+$ T$_{RM}$ cells (ASYMP HLA-Tg rabbits)			Gene expression in CD8$^+$ T cells (ASYMP HLA-Tg rabbits)		
**Gene**	**FC**	***P*-value**	**FDR**	**FC**	***P*-value**	**FDR**
JAK2	2.02	0.009	0.02	2.30	0.009	0.02
CCL17	2.05	0.03	0.05	5.25	1.60E-07	8.55E-06
CCL20	3.01	0.01	0.05	3.66	0.001	0.01
CCL22	4.20	0.002	0.01	4.45	3.07E-05	0.0006
CCL4	3.01	9.65E-10	7.99E-07	2.35	3.53E-13	8.30E-11
CDC42	2.51	0.02	0.04	2.41	0.0001	0.001
CHUK	2.02	0.009	0.02	2.75	0.0008	0.006
CX3CR1	3.73	0.0001	0.005	2.47	3.36E-09	2.82E-07
CXCL10	3.60	0.01	0.05	2.08	0.009	0.02
CXCL8	3.03	0.004	0.03	2.50	3.89E-05	0.0006
CXCR4	4.58	0.001	0.04	4.33	1.92E-26	1.62E-23
GNB1	3.22	0.0005	0.02	3.05	3.92E-05	0.0007
GRK3	2.06	0.0009	0.02	8.30	2.17E-09	1.95E-07
CCL14	3.02	0.03	0.05	2.98	8.84E-05	0.001
PAK1	2.56	0.02	0.05	2.50	0.01	0.05
PTK2B	2.13	0.001	0.04	4.88	0.0004	0.006
TIAM1	3.03	0.02	0.04	3.22	0.003	0.01
CXCR3	2.63	0.003	0.02	4.52	0.009	0.05
STAT5B	4.06	0.01	0.05	3.03	0.02	0.05
GRB2	3.10	0.01	0.02	2.03	0.004	0.02
GNB2	2.70	0.04	0.06	4.17	0.006	0.02
CCR7	5.98	2.09E-07	7.52E-05	4.57	0.01	0.05
CXCL11	2.93	0.002	0.01	3.21	0.02	0.05
CCL5	3.29	4.98E-30	4.13E-26	2.08	0.005	0.01

CXCL9	2.01	0.03	0.05	4.60	0.003	0.02
RAP1B	3.59	0.004	0.03	3.30	0.009	0.04
BRAF	2.96	0.004	0.01	2.77	0.01	0.05
RAC2	-1.05	0.008	0.01	-0.85	0.02	0.05
RAF1	-2.04	0.0001	0.002	-1.45	0.001	0.01
RAP1A	-0.06	0.008	0.04	-0.96	0.0006	0.007
ROCK1	-0.41	0.02	0.05	-1.07	0.006	0.03
CRKL	-2.40	0.03	0.05	-0.52	0.01	0.06
KRAS	-1.01	0.01	0.05	-6.29	0.007	0.03

FC: Fold change gene expression; Statistical significance when $P \leq 0.05$; FDR: False discovery rate.

The differential gene expression data shown for CD8$^+$ T_{RM} cells are based on the CD8$^+$ T_{RM} specific normalized gene count data obtained from single-cell RNA sequencing. The CD8$^+$ T cell specific differential gene expression for HLA-Tg rabbits were based on the normalized gene count data obtained from Bulk-RNA sequencing. The comparison was made between ASYMP vs SYMP groups.

Supplementary Table S4. Cytokine-cytokine receptor interaction pathway specific genes differentially expressed in the CD8$^+$ T$_{RM}$ cells, and CD8$^+$ T cell in rabbit Trigeminal ganglia

	Gene expression in CD8$^+$ T$_{RM}$ cells (ASYMP HLA-Tg rabbits)			Gene expression in CD8$^+$ T cells (ASYMP HLA-Tg rabbits)		
**Gene**	**FC**	***P*-value**	**FDR**	**FC**	***P*-value**	**FDR**
CD4	2.95	0.004	0.03	2.85	0.01	0.05
IFNG	2.03	0.009	0.01	2.19	0.01	0.05
IFNGR1	3.08	0.0008	0.004	7.15	0.0001	0.001
IFNGR2	4.00	0.05	0.68	2.42	0.0009	0.009
IL12RB2	4.03	0.001	0.03	9.18	1.99E-14	6.15E-12
IL13RA1	2.36	0.008	0.04	5.74	0.02	0.05
IL15RA	2.60	0.0002	0.008	2.51	0.001	0.01
IL16	2.06	0.002	0.03	2.18	0.0001	0.002
IL17RA	3.02	0.004	0.01	2.13	0.0008	0.008
IL17RC	1.03	0.01	0.05	6.93	0.0003	0.003
IL1B	2.90	0.009	0.03	3.19	0.02	0.06
IL1R1	4.03	0.01	0.05	5.61	9.30E-14	2.46E-11
IL1RAP	3.05	0.008	0.01	7.59	2.48E-06	7.04E-05
IL23A	2.02	0.005	0.03	4.57	0.0001	0.001
IL2RG	6.09	0.006	0.04	5.71	0.009	0.05
IL6	2.20	8.77E-07	0.0002	5.65	7.97E-24	6.32E-21
IL6ST	6.57	0.001	0.03	6.69	0.005	0.04
IL7R	5.77	0.0002	0.02	4.35	4.50E-09	4.13E-07
LEPR	4.02	0.004	0.05	3.38	1.18E-05	0.0003
LTA	4.80	0.003	0.02	5.80	0.01	0.05
TGFBR1	3.00	0.005	0.01	2.44	1.53E-08	1.21E-06
TNFR2	2.89	0.007	0.03	3.90	0.001	0.01
CD137	7.16	0.01	0.05	2.35	6.84E-15	2.17E-12
HVEM	4.84	0.004	0.02	3.12	0.009	0.04

OSMR	-3.60	0.0008	0.001	-5.91	0.01	0.05
IL1A	-1.02	0.002	0.03	-3.27	0.0008	0.007
IL10RB	-2.07	0.0007	0.002	-2.30	0.004	0.05
IFNAR1	-0.04	3.72E-05	0.0007	-0.92	0.006	0.04
IFNAR2	-1.02	0.0004	0.002	-6.49	0.001	0.01

FC: Fold change gene expression; Statistical significance when $P \leq 0.05$; FDR: False discovery rate.

The differential gene expression data shown for $CD8^+$ T_{RM} cells are based on the $CD8^+$ T_{RM} specific normalized gene count data obtained from single-cell RNA sequencing. The $CD8^+$ T cell specific differential gene expression for HLA-Tg rabbits were based on the normalized gene count data obtained from Bulk-RNA sequencing. The comparison was made between ASYMP vs SYMP groups.
