# Supplementary material for: Unique molecular signatures of antiviral memory CD8+ T cells associated with asymptomatic recurrent ocular herpes
Source: Sci Rep. 2020 Aug 14;10:13843. doi: 10.1038/s41598-020-70673-z (PMC7427992; doi:10.1038/s41598-020-70673-z)
Supplement: Supplementary file 4 — Supplementary Legends. [file 41598_2020_70673_MOESM4_ESM.docx]

**Supplementary Figure S1: Differential genes expression in HSV-specific CD8^+^ T cells from HSV-1 infected symptomatic vs. asymptomatic individuals and trigeminal ganglia of HSV-1 infected symptomatic vs. asymptomatic HLA Tg rabbits:** (**a**) The Venn diagram shows the number of up-regulated (583) and down-regulated (189) human genes out of a pool of 772 genes (*top panels*). Genes showing a minimum count per million (CPM) ≥ 0.5 were used to obtain the transformed counts data for clustering using regularized log (rlog). The correlation matrix shows the degree of relatedness among the SYMP and ASYMP individuals (*middle panels*). Pearson's correlation coefficient was applied to compute the correlation matrix. The Volcano plot showing the log2 fold change and -log10 (FDR adjusted *P*-value) of each of the differentially expressed genes in HSV-specific CD8^+^ T cells from SYMP vs. SYMP individuals (*lower panels*). (**b**) The Venn diagram shows the numbers of upregulated (1,605) and downregulated (1,274) rabbit genes out of a pool of 2,879 genes (*top panels*). The correlation matrix shows the degree of relatedness among the SYMP and ASYMP HLA Tg rabbits (*middle panels*). Pearson's correlation coefficient was applied to compute the correlation matrix. The Volcano plot showing the log2 fold change and -log10 (*P*-value) of each of the differentially expressed genes in HSV-specific CD8^+^ T cells from SYMP vs. SYMP HLA Tg rabbits (*lower panels*).

**Supplementary Figure S2: Gating strategy used for FACS sorting of Tetramer specific CD8^+^ T cells:** Lymphocytes were first selected from the whole human PBMC by plotting low forward scatter (FSC-A) versus low side scatter (SSC-A). After that, the singlets were identified by forward scatter area (FSC-A) and forward scatter height scatter (FSC-H) gate. The CD3 positive cells were then identified and gated by the expression of the CD3 antibody. Then CD8 positive cells were identified and gated by the expression of CD8 antibody. Finally, we gated on HSV specific CD8+ T cell for the sorting using the gB_561-569_ and VP11-12_702-710_ tetramers. Right after the sorting, post sorting purity was assessed by FACS acquisition of few sorted cells.
